# Death of parent, sibling, spouse, and child in a Swedish national sample and risk of subsequent stress reaction, major depression, alcohol-use disorder, and drug-use disorder

**DOI:** 10.1017/S0033291723000570

**Published:** 2023-11

**Authors:** Kenneth S. Kendler, Sara L. Lönn, Jan Sundquist, Kristina Sundquist

**Affiliations:** 1 Virginia Institute for Psychiatric and Behavioral Genetics, Virginia Commonwealth University, Richmond, VA, USA; 2 Department of Psychiatry, Virginia Commonwealth University, Richmond, VA, USA; 3 Center for Primary Health Care Research, Lund University, Malmö, Sweden; 4 Department of Family Medicine and Community Health, Department of Population Health Science and Policy, Icahn School of Medicine at Mount Sinai, New York, NY, USA; 5 Center for Community-based Healthcare Research and Education (CoHRE), Department of Functional Pathology, School of Medicine, Shimane University, Matsue, Japan

**Keywords:** Alcohol-use disorder, drug-use disorder, major depression, stress disorder, stressful life events

## Abstract

**Background:**

To determine, in a general population, how much rates of stress reactions (SR), major depression (MD), alcohol-use disorder (AUD) and drug-use disorder (DUD) increase after the death of close relatives.

**Methods:**

SR, MD, AUD, and DUD registrations were assessed from national Swedish registries. From the population followed from 2000 to 2018, those exposed to death of a close relative in 2002–2016 were matched to unexposed controls and analyzed in males and females by a controlled pre-post design using a difference-in-difference method.

**Results:**

Substantial, brief increases in risk for SR and more modest prolonged increases in MD were observed after death of relatives in both men and women greatest with children, followed by spouses, parents, and siblings. Relatively long-lasting modest increases in AUD but not DUD were also observed following death of relatives. The absolute increases for SR and MD were greater in females than males and for AUD greater in males than females. However, logistic regression analyses showed most effects did not differ significantly by sex. Consistently larger increases in disorder risk were seen with the death of younger *v.* older parents, siblings, and spouses and with accidental *v.* non-accidental death in children.

**Conclusions:**

Applying a matched cohort design to Swedish population registries, death of close relatives was associated with, and likely caused, substantial increases in rates of SR, MD, and AUD, consistent with smaller prior clinical investigations. Through such registries, we can, in large representative samples, integrate the impact of exposures to selected environmental adversities into disorder risk pathways.

A long tradition of research has examined the association between stressful life events (SLEs) and risk for episode onset or recurrence of psychiatric disorders, especially major depression (MD) (Brown & Harris, 1978; Brown, Harris, & Hepworth, 1995; Cohen, Murphy, & Prather, 2019; Holmes & Rahe, 1967; Kendler, Karkowski, & Prescott, 1998; Kessler, 1997; Paykel et al., 1969). A substantial proportion of this association appears to be causal (Kendler & Gardner, 2010a; Kendler, Karkowski, & Prescott, 1999). Most, but not all, studies have also seen elevations in rates of alcohol use and alcohol-use disorder (AUD) (Boden, Fergusson, & Horwood, 2014; Gorman & Peters, 1990; Jennison, 1992; Keyes, Hatzenbuehler, & Hasin, 2011; Lee, Young Wolff, Kendler, & Prescott, 2012; Perreira & Sloan, 2001; Storr et al., 2021) and drug use and drug-use disorder (DUD) (Hyman & Sinha, 2009; Myers, McLaughlin, Wang, Blanco, & Stein, 2014) after SLE exposure. Nearly all such studies have relied on questionnaire or interview-based retrospective assessments of SLEs with the more detailed interview-based measures recognized as producing more valid measures (Brown, Sklair, Harris, & Birley, 1973; Dohrenwend, Dohrenwend, Dodson, & Shrout, 1984; Dohrenwend, Link, Kern, Shrout, & Markowitz, 1987; Dohrenwend, Raphael, Schwartz, & Skodol, 1993; Paykel, 1983). However, such assessments are time consuming and thus typically available on only limited samples. Furthermore, problems of interpretation remain including accurate timing of events, memory biases that might favor event recall when followed by illness onset and reverse causation in which the disorder predisposes to selection into stressful environments.

In this report, we evaluate a different methodological approach to SLE research that has only rarely been employed (Kessing, Agerbo, & Mortensen, 2004; Li, Tsai, Chen, Liang, & Chen, 2022): the use of national registries. We examine changes in the rates of registration, in national medical and criminal registries, of four psychiatric diagnoses [stress reactions (SR), MD, AUD, and DUD] before and after the death of a parent, sibling, spouse, or child. As an SLE, death in a close relative has the methodological advantages of being temporally discrete, non-recurrent, assessed with high accuracy, and reflecting a severe stressor (Brugha, Bebbington, Tennant, & Hurry, 1985; Holmes & Rahe, 1967; Kendler et al., 1998). Furthermore, prior work based on measures of grief intensity provide us with predictions that can validate our approach – that psychiatric reactions to death are more severe when the death (i) occurs unexpectedly (Ball, 1977; Keyes et al., 2014; Shear, 2015), (ii) involves children compared to other close relatives (Leahy, 1993; Middleton, Raphael, Burnett, & Martinek, 1998; Sanders, 1980; Shear, 2015), and (iii) occurs in adult relatives prematurely, that is in young or middle age compared to late adult life (Ball, 1977; Segal & Bouchard, 1993). To maximize our ability to infer causal effects from our observational data, we utilize a controlled pre-post design analyzed using a difference-in-difference method (Lechner, 2010; Ohlsson & Kendler, 2020).

We examine rates of registration for SR, MD, AUD, and DUD in exposed cases *v.* unexposed controls for 2 years before and after the death of a parent, sibling, spouse, or child. We evaluate the degree to which the pattern differs across disorders, sexes, and category of deceased relative. We then examine the validity of our method by comparing differences in rates of disorder in a matched cohort design as a function of the age of the deceased parent, sibling, or spouse and, for children, whether the death was accidental or not.

## Methods

We linked nationwide Swedish registers via the unique 10-digit identification number assigned at birth or immigration to all Swedish residents. The identification number was replaced by a serial number to ensure confidentiality. For the sources used to create our dataset, see online Appendix Table 1.

We identified males and females who were exposed to the death of a close relative between 2002 and 2016. If exposed to death of a parent, sibling, or spouse, we constrained the population to individuals exposed after age 18. For death of child, we required that the child was under age 18. We only examined cases with the first death in each relative class. Using Swedish hospital, specialistic and primary care medical registers, which contain ICD codes for each hospital discharge and out-patient medical contact, we defined SR using ICD-10 code F43 and MD using ICD-10 codes: F32 and F33 and ICD-9 codes: 296B, 298A, and 300E. For the definitions of AUD and DUD, see online Appendix Table 2.

### Statistical methods

**Fig. 1.**
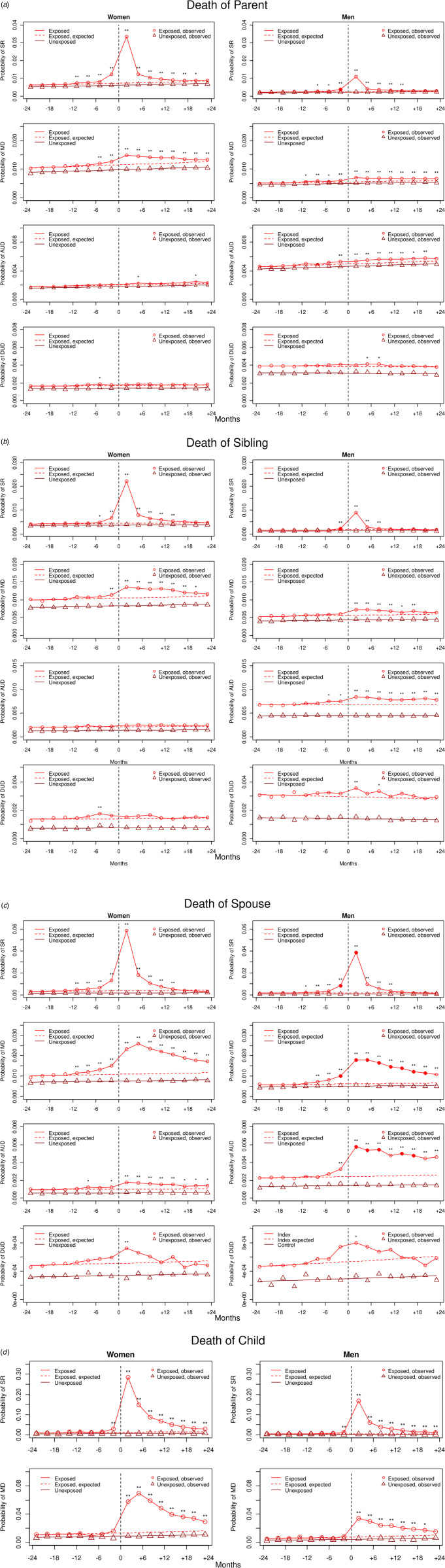
(*a*) Rates of registration for SR, MD, AUD, and DUD in male and female exposed cases and unexposed controls 2 years before and 2 years after the death of a *parent* of an exposed case. The *x*-axis represents the 24 months before (−) and the 24 months after (+) parental death. The zero point from which a dotted vertical black line emerges, represents the date of death in the parent of the exposed case. The *y*-axis represents the probability of registration for that disorder in the three months period. The solid red/black line and circles represent the estimated and observed rates, respectively, of disorder in the exposed individuals. Correspondingly, the brown/gray line and triangles represent the estimated and observed rates of the disorder in the unexposed individuals. The dotted red/black line is the extrapolated rates of illness in the exposed cases expected from the results of the exposed and unexposed individuals for the first 12 months of observation. Asterisks above the solid red/black line with circles indicate the presence of significant differences in the ORs in exposed *v.* unexposed individuals: *p < 0.01 and **p < 0.001. Significant differences in the exposed-unexposed disorder ORs between males and females (*p* < 0.01) are represented by filled in red/black circles in the results for males. See online Appendix Table 5 for numerical estimates of all the exposed-unexposded ORs with 95% CIs and differences between those ORs in males *v.* female. (*b*) Rates of registration for SR, MD, AUD, and DUD in male and female exposed and unexposed individuals 2 years before and 2 years after the death of a *sibling* of an index case. For other details, see legend to (*a*). (*c*) Rates of registration for SR, MD, AUD, and DUD in male and female exposed and unexposed 2 years before and 2 years after the death of a *spouse* of an exposed individual. For other details, see legend to (*a*). (*d*) Rates of registration for SR, MD, AUD, and DUD in male and female exposed and unexposed 2 years before and 2 years after the death of a *child* of an exposed case. For other details, see legend to (*a*).

**Fig. 2.**
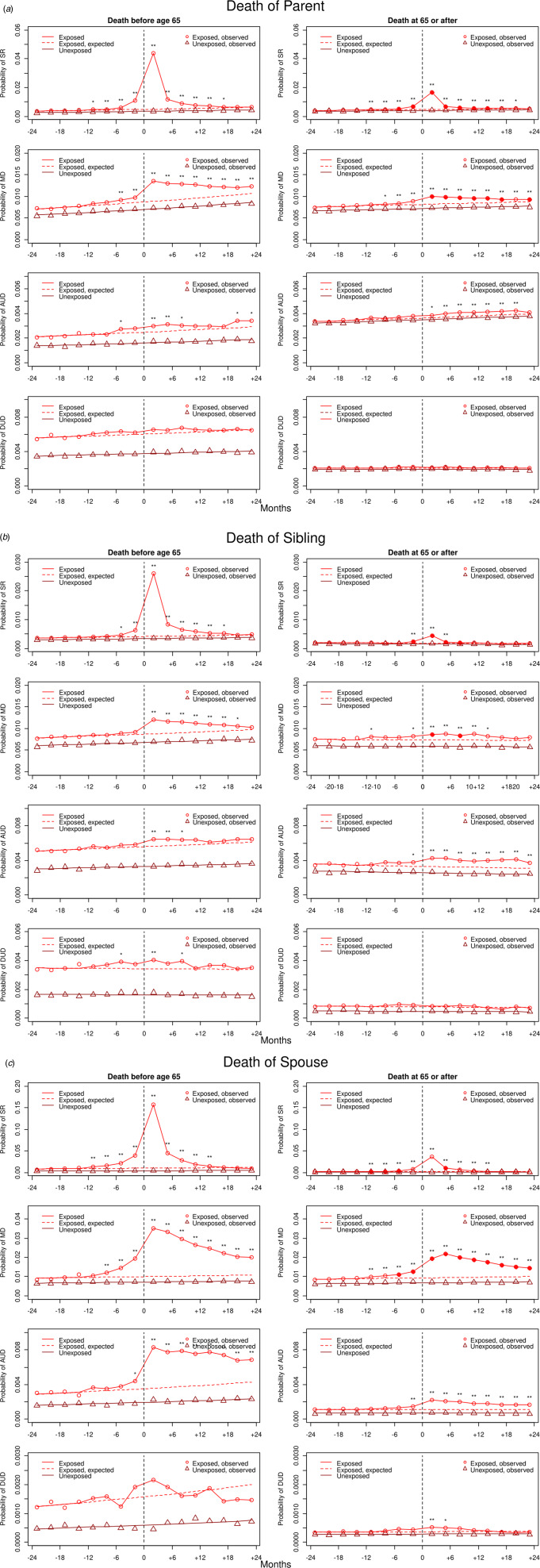
(*a*) Rates of registration for SR, MD, AUD, and DUD in exposed and unexposed individuals 2 years before and 2 years after the death of a *parent* of an index case at younger than age 65 *v.* at age 65 or older. The *x*-axis represents the 24 months before (−) and the 24 months after (+) parental death. The zero point from which a dotted vertical black line emerges, represents the date of death. The *y*-axis represents the probability of registration for that disorder in the prior 3 months. The solid red/black line with circles represents the rates of disorder in the exposed individuals and the brown/gray line with triangles in those that are unexposed. The dotted red/black line is the extrapolated rates of illness in the exposed individuals expected from the results of the exposed and unexposed for the first 12 months of observation. Asterisks above the solid red/black line with circles indicate the presence of significant differences in the ORs in exposed *v.* unexposed: *p < 0.01 and **p < 0.001. Significant differences in the exposed-unexposed ORs between individuals when the parent died <65 *v.* ≥65 (*p* < 0.01) are represented by filled in red/black circles in the results for those dying at ≥65. See online Appendix Table 6 for numerical estimates of all the exposed-unexposed ORs with 95% CIs and differences between those ORs in exposed individuals who lost relatives at a younger *v.* older age. (*b*) Rates of registration for SR, MD, AUD, and DUD in exposed and unexposed 2 years before and 2 years after the death of a *sibling* of an exposed individual at younger than age 65 *v.* at age 65 or older. Significant differences in the exposed-unexposed ORs between individuals when the sibling died <65 *v.* ≥65 (*p* < 0.01) are represented by filled in red/black circles in the results for those dying at ≥65. For other details, see legend to (*a*). (*c*) Rates of registration for SR, MD, AUD, and DUD in exposed and exposed individuals 2 years before and 2 years after the death of a *spouse* of an exposed individual at younger than age 65 *v.* at age 65 or older. Significant differences in the exposed-unexposed ORs between exposed individuals when the sibling died <65 *v.* ≥65 (*p* < 0.01) are represented by filled in red/black circles in the results for those dying at ≥65. For other details, see legend to (*a*).

**Fig. 3.**
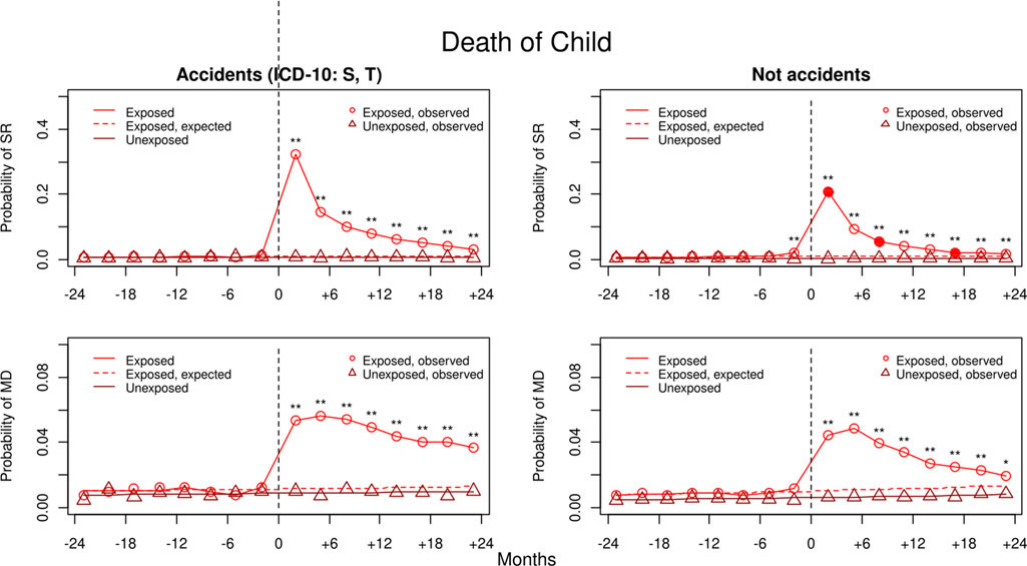
Rates of registration for SR and MD in exposed and unexposed individuals 2 years before and 2 years after the death of a *child* from accidental and non-accidental causes. The *x*-axis represents the 24 months before (−) and the 24 months after (+) parental death. The zero point from which a dotted vertical black line emerges, represents the date of death. The *y*-axis represents the probability of registration for that disorder in the prior 3 months. The solid red/black line with circles represents the rates of disorder in the exposed individual and the brown/gray line with triangles the unexposed. The dotted red/black line is the extrapolated rates of illness in the exposed individuals expected from the results of the exposed and unexposed for the first 12 months of observation. Asterisks above the solid red/black line with circles indicate the presence of significant differences in the ORs in exposed *v.* unexposed: *p < 0.01 and **p < 0.001. Significant differences in the exposed-unexposed ORs between exposed individuals when the child died from accidental *v.* non-accidental causes (*p* < 0.01) are represented by filled in red/black circles in the results for those dying of non-accidental causes. See online Appendix Table 6 for numerical estimates of all the exposed-unexposed ORs with 95% CIs and differences between those ORs in individuals who lost a child from an accidental *v.* non-accidental death.

In our matched cohort design, we compare the relative increase in risk for the *exposed v.* an *unexposed* group (Sjölander & Greenland, 2013). For our matching variables, see online Appendix Table 3. We next identified affected individuals in 3-month periods, before and after the relative death, comparing the exposed to the unexposed utilizing logistic regression in which the log odds depending linearly on time. For the exposed group, the time-period 2 to 1 year before the death is defined as baseline. During this time period, our model assumes a constant difference between the exposed and unexposed. One year before the event we estimate the increased risk, compared to the expected, in 3 month periods by including dummy variables for each time period. Our parameter estimates the increase in odds compared to what would have been expected if the exposed group would have had the same difference in risk compared to the unexposed group during the baseline period. In addition, the model, which is presented in online Appendix Table 4, allows for separate associations in males and females. We present the increase in risk for the exposed *v.* unexposed individuals as odds ratio (OR) with 95% confidence intervals (CIs), together with a graphical illustration of the probabilities for the unexposed and exposed. The expected probabilities in the unexposed are represented by a dashed line. Because of the large number of tests, we only indicate the 1% and 0.1% significances in the figures. The significance of the sex differences or age of the death of the relative is not presented but instead indicated by filled in triangles in the figures representing men. Our main results are presented in Figs 1–3 which are also given with 95% CIs in online Appendix Tables 5 and 6.

Thereafter, we investigated if the age of the relative, or cause of death of the child could have an impact on the increase in risk. We divided the parents, siblings, and spouses into two groups: younger or older than 65 and the age of retirement in Sweden. Children were divided into those who died accidently, defined by ICD-10 as S or T diagnosis, and those who did not.

## Results

The number of exposed cases in our sample who had a parent, sibling, spouse, or child who had died during our observation period varied widely, and was highest for parents, followed by spouses and siblings with much smaller numbers for children, which made it impossible to usefully compare rates of AUD and DUD in their parents (Table 1). Table 1 also provides the raw rates of SR, MD, AUD, and DUD in the two years before and after the death in our exposed and matched unexposed controls. In all figures, the significance of the differences, as assessed by ORs, are marked by asterisks above each result for cases while differences between the sexes or the age of the deceased are noted by solid triangles. Each circle represents the observed rate of illness over the preceding 3 months so that the circle at +3 months reflects rates for the first 3 months after the death of a relative. For further details, see figure legends.

**Table 1.** Descriptive results for death of close relative in matched case and control samples

			Number of registrations and prevalence							
			SR		MD		AUD		DUD	
	Relative	Sample size	*N*	%	*N*	%	*N*	%	*N*	%
Females										
Exposed, before	Sibling	181 924	4544	2.50	7913	4.35	1494	0.82	1086	0.60
Exposed, after			7497	4.12	9326	5.13	1795	0.99	1139	0.63
Unexposed, before			3467	1.91	6342	3.49	1040	0.57	598	0.33
Unexposed, after			3558	1.96	6564	3.61	1076	0.59	582	0.32
Males										
Exposed, before	Sibling	178 016	1682	0.94	4235	2.38	4897	2.75	1934	1.09
Exposed, after			3000	1.69	5088	2.86	5573	3.13	1964	1.10
Unexposed, before			1267	0.71	3297	1.85	3244	1.82	1038	0.58
Unexposed, after			1280	0.72	3430	1.93	3259	1.83	984	0.55
Females										
Exposed, before	Spouse	241 994	7184	2.97	12 559	5.19	1099	0.45	564	0.23
Exposed, after			18 564	7.67	21 735	8.98	1501	0.62	656	0.27
Unexposed, before			1944	0.80	8384	3.46	589	0.24	377	0.16
Unexposed, after			2290	0.95	9102	3.76	637	0.26	416	0.17
Males										
Exposed, before	Spouse	108 011	1781	1.65	3505	3.25	1150	1.06	256	0.24
Exposed, after			5261	4.87	6966	6.45	2168	2.01	332	0.31
Unexposed, before			343	0.32	2546	2.36	674	0.62	160	0.15
Unexposed, after			381	0.35	2735	2.53	711	0.66	161	0.15
Females										
Exposed, before	Parent	557 970	21 184	3.80	24 406	4.37	4128	0.74	3643	0.65
Exposed, after			35 826	6.42	30 044	5.38	4794	0.86	3872	0.69
Unexposed, before			15 671	2.81	21 069	3.78	3725	0.67	3020	0.54
Unexposed, after			17 960	3.22	22 944	4.11	4175	0.75	3170	0.57
Males										
Exposed, before	Parent	590 045	8046	1.36	13 011	2.21	11 540	1.96	8402	1.42
Exposed, after			13 649	2.31	15 935	2.70	12 999	2.20	8444	1.43
Unexposed, before			6565	1.11	11 926	2.02	10 654	1.81	6846	1.16
Unexposed, after			7198	1.22	12 976	2.20	11 524	1.95	6823	1.16
Females										
Exposed, before	Child	5845	420	7.19	277	4.74				
Exposed, after			2109	36.08	927	15.86				
Unexposed, before			170	2.91	201	3.44				
Unexposed, after			217	3.71	230	3.93				
Males										
Exposed, before	Child	5849	190	3.25	164	2.80				
Exposed, after			1226	20.96	515	8.80				
Unexposed, before			73	1.25	88	1.50				
Unexposed, after			87	1.49	126	2.15				

### Main findings

#### Parents

Figure 1*a* shows the risk for diagnoses of SR, MD, AUD, and DUD 2 years before and after a parental death, separately in males and female. In both men and women, rates of SR peak at +3 months. We see a much smaller absolute rise of risk in males *v.* females but because of differences in base rates, the ORs at +3 months were similar: 4.70 (4.58–4.82) in females and 4.52 (4.34–4.71) in males.

The increased risk for SR then declines rapidly in both sexes but remains significant for over 1 year. Both sexes show small but significant increases in SR rates in the year preceding the death. The only significant sex difference was an OR higher in females in the 3 months prior to parental death.

More modest increases in rates of MD are seen in both sexes beginning in the year prior to the death with a much slower decline than seen with SR. While the absolute rise is greater in females than males, no significant differences were seen in the observed ORs which maximized at +3 months in both sexes: 1.30 (1.26–1.33) in females and 1.30 (1.23–1.36) in males.

Rates of AUD had no sustained increase in risk in females after parental death while we see a modest significant increase in rates in men over most of the 2-year follow-up period with a maximal OR of 1.10 (1.06–1.15) at +9 months. For DUD, we see no sustained increase associated with parental death in either sex but a small but significant increase at +6 and +9 months.

#### Siblings

As seen in Fig. 1*b*, the prevalence for our four disorders after death of a sibling in males and females closely resembles that seen in parents with (i) a sharp rise of risk for SR with ORs of 5.16 (4.89–5.45) in females and 5.35 (4.89–5.84) in males that declines quickly, (ii) a slower more sustained rise in risk for MD with ORs maximizing at 1.30 (1.23–1.36) in females and 1.28 (1.20–1.37) in males, (iii) a sustained modest significant increased risk for AUD in males [peak OR 1.24 (1.17–1.32)] but not females, and (iv) no durable increase in either sex for DUD. As with death of parent, the absolute level of increase for SR and MD was substantially greater in females, the ORs of the two sexes differed significantly only at one time point (−3 months for SR).

#### Spouse

The increase in risk for SR and MD after death of spouse is substantially greater than that observed after the death of a parent or sibling. A sharp rise of SR starts at −3 months, maximizing at +3 months. While the absolute rates at both time points are quantitatively greater in females than males, the ORs are significantly higher in males than females. At +3 months, these equal to, respectively [24.19 (21.03–27.82)] and [18.34 (15.98–21.04)]. Rates of SR then decline rapidly.

The rise of risk for MD also begins well before the spousal death, is slower in its rise and decline and of more modest magnitude than seen with SR. The ORs are significantly greater in males than females for 2 years – from −3 to +21 months. For example, at +3 months, the ORs are respectively, 3.08 (2.86–3.33) and 2.31 (2.13–2.52).

Both males and females demonstrate a significantly increased risk for AUD after death of their spouses lasting throughout our observation period. The absolute value of the increase in AUD risk is consistently greater in men than women and the ORs are significant greater in men over nearly the entire follow-up period. For both sexes, the risk is highest at +3 months and equals 1.79 (1.57–2.03) in females and 2.45 (2.18–2.74) in males. Both sexes demonstrate a short-lived modest significantly increased risk for DUD after spousal death.

#### Child

The increases in risk for SR and MD after death of a child were substantially higher than those observed with deaths of parents, siblings, or spouses. For SR, we see a sharp and very large increase in mothers maximizing at +3 months with an OR of 31.3 (26.17–37.43). The absolute increase in males was similar in shape but smaller while the ORs at +3 months were non-significantly larger: 42.26 (32.37–55.16).

For MD, we see, in females, a more slowly rising peak maximizing at +6 months with an OR of 5.27 (4.35–6.38) and a subsequent fall off in risk over the follow-up period. For males, the risk rise was smaller in magnitude and maximized at +3 months with an OR of 4.51 (3.49–5.82). The ORs did not differ significantly across females and males for either SR or MD.

### Validation

The increased risks for both SR and MD were substantially and significantly reduced in individuals after the death of a parent, sibling, or spouse who was 65 or older *v.* under age 65 (Fig. 2*a*–2*c*).

Rates of AUD were appreciably lower after the death of an older *v.* younger spouse, but these resulting ORs were not significantly different at our 1% threshold. The rates of both SR and MD were lower for parents who lost their child from non-accidental *v.* accidental deaths, but only the differences in SR were statistically significant.

## Discussion

We sought, in these matched cohort pre-post analyses, to examine the association between death of close relatives and rates of registration for four psychiatric diagnoses in the Swedish general adult population. Furthermore, we sought to determine differences in the magnitude of the changes in rates across (i) disorders, (ii) class of relative, and (iii) the sex of the exposed case. We review these in turn.

We observed systematic differences in the rates of our four disorders immediately prior to and after the death of a relative that were stable across the kind of deceased relative and the sex of the exposed individual. SR had the most immediate and largest rise in rates and, typically, the most rapid decline. MD showed a slower and smaller increase in risk that tended to be longer-lasting. When present, the increase of AUD was modest to moderate in magnitude but persistent. Finally, we saw little evidence of a sustained significant increase in DUD after the death of a close relative.

In most of our analyses, the rise in disorder risk preceded the death of the relative, particularly in spouses. This rise likely resulted from several causes including severe illnesses, injuries, and care-giving burdens that were stressful for the exposed case as well as anticipatory loss. As predicted by this hypothesis, no elevation in the rates of SD and MD were seen prior to accidental death of children.

We also found large differences in the magnitude of the increase in disorder after the death of our four classes of relatives. The peak ORs for SR in women after the death of a parent, sibling, spouse, and child, equaled, respectively, 4.70, 5.16, 16.71, and 31.30. The maximal OR for MD after the death of these four classes of relatives in males equaled 1.27, 1.28, 2.97, and 4.51, respectively. Our results, particularly with respect to rates of SR and MD, are consistent with the prior literature on grief intensity, suggesting that on average, in adulthood, the affective bonds and resulting intensity of bereavement on loss, are strongest with children, followed by spouses, and then parents and siblings (Leahy, 1993; Middleton et al., 1998; Sanders, 1980; Shear, 2015).

For males, the maximum OR for AUD was considerably higher after the death of a spouse (2.45) than after the death of a parent or sibling (1.10, 1.24), with a similar pattern of ORs seen in females: 1.79, 1.12, and 1.16. The much greater risk in AUD risk after spousal death may arise because, in addition to the emotional stress of the loss, the death of a spouse also means the disappearance of the well-established protective effect of the presence of spouses against rates of risky drinking and alcohol abuse (Leonard & Rothbard, 1999; Umberson, 1992). Indeed, in Sweden that state of marriage is substantially protective against risk for AUD (Kendler, Lonn, Salvatore, Sundquist, & Sundquist, 2016a) while divorce substantially increases AUD risk (Kendler, Lonn, Salvatore, Sundquist, & Sundquist, 2017).

The interpretation of our findings on sex differences in the rates of disorders after death of close relatives is a function of the scale of measurement for which there is no right or wrong answer (Kendler & Gardner, 2010b; Rothman, Greenland, & Lash, 2008). Assuming an additive model, we see, after death in a close relative, substantially greater increases in rates of SR and MD in women *v.* men and AUD in men *v.* women. However, if we assume a multiplicative scale of measurement, as assessed by ORs, we see relatively few sex differences, the most striking of which is the greater rise in rates of MD and AUD in males *v.* females after the death of their spouse. These results may be due to the greater mental health marriage benefit obtained by men *v.* women (Kiecolt-Glaser & Newton, 2001; Umberson, 1992).

We next validated our method by showing, consistent with prior evidence that the psychological reaction to death in adult relatives is stronger if the death occurs prematurely (24, 29), that rates of SR and MD were significantly higher when parents, siblings and spouses died under *v.* over age 65. In agreement with prior studies, we also showed higher rates of SR and MD in parents when their children died by accidental *v.* non-accidental causes (Ball, 1977; Keyes et al., 2014; Shear, 2015).

Finally, we combined a pre-post and matched cohort design analyzed by a difference-in-difference method to maximize our power for causal inference (Ohlsson & Kendler, 2020). While we cannot rule out a role for hidden confounders, the consistency of our findings and the power of our statistical approach suggest that the observed associations between increased rates of SR, MD, and AUD and the death of close relatives are largely causal in nature.

Our results have one straight forward clinical implication. Following the death of a close relative, survivors are at high risk for a range of psychiatric outcomes. Brief interventions by health professionals may help to insure these conditions are not sustained and impairing in the future.

### Limitations

Six potentially important limitations of this work should be considered. First, its value depends on the quality of diagnoses in Swedish medical registries, which has been rather widely studied and supported (Ludvigsson et al., 2011). The validity of MD diagnoses is supported by its prevalence, sex ratio, sibling, and twin correlations and associations with well-documented psychosocial risk factors (Kendler, Ohlsson, Lichtenstein, Sundquist, & Sundquist, 2018; Sundquist, Ohlsson, Sundquist, & Kendler, 2017). We have also shown that the genetic profile of cases of MD is similar for those ascertained in primary care, specialist, and in-patient settings (Kendler, Ohlsson, Bacanu, Sundquist, & Sundquist, 2022). The validity of our definitions of AUD and DUD are reinforced by the high rates of concordance for ascertainment across registries (Kendler et al., 2012, 2015), and the similarity of genetic epidemiological findings for AUD and DUD in Sweden compared to those in other samples (Kendler et al., 2012, 2015, 2016b; Kendler, Maes, Sundquist, Ohlsson, & Sundquist, 2013). We know of no work evaluating the validity of SR diagnoses in Sweden. We explored the effects of death of relatives on the SR ICD subtypes (see online Appendix Table 7) and found significant elevations in rates in exposed *v.* unexposed individual across all subtypes, being highest for F430 (acute stress reaction) and F439 (Reaction to severe stress, unspecified) and lowest in F438 (Other reactions to severe stress).

Second, we could only study cases of SR and MD coming to clinical attention (while cases of AUD and DUD were also detected through criminal registries). While Sweden has a nationalized medical system with minimal barriers to care, it is likely that a proportion of individuals with SR or depressive syndromes did not seek medical care. The ORs we found for death of relatives and MD, which ranged from 1.27 for siblings of males to 5.27 for children of females, are generally lower than those obtained from retrospective interview-based studies (e.g. Kendler et al., 1998). This discrepancy could have resulted from a downward bias in our estimates due to missing untreated cases and/or upward biases from selective recall and/or mood-biased recall (Bower, 1981). However, an upward bias on our ORs might arise if death of a relative increased physician attendance of the exposed case, thereby providing chances for a diagnosis of SR, MD, AUD, or DUD.

Third, while our main analyses did not distinguish recurrent from incident cases, we present such results in online Appendix Figs 1–4. The main patterns of our findings are seen in both subgroups, although, the estimated effects are generally larger in incident *v.* recurrent cases. This is likely due to part to the much lower incidence rates for our disorders in the unexposed controls.

Fourth, while death is typically a temporally discrete event, the process of dying can differ widely across individuals and present to their relative's variable stressors and care-giving burdens. Evidence for this is seen in the frequent elevation of rates of our disorders in exposed cases preceding the death of their relative.

Fifth, while our assessment of the death of relatives is objective and accurately dated, our registry data do not permit us to assess a number of dimensions of the loss that require respondent report, such as long-term contextual threat developed by Brown which has been shown to robustly predict rates of subsequent depressive episodes (Brown & Harris, 1989; Kendler et al., 1998).

Finally, in examining potential psychiatric syndromes after the death of loved ones, concern is appropriate regarding discrimination between a psychiatric illness and normal grief. In using diagnoses coded by thousands of Swedish physicians, we have no control over this diagnostic distinction. This concern is greatest for SR which are described in ICD-10

…as maladaptive responses to severe or continued stress, in that they interfere with successful coping mechanisms and therefore lead to problems of social functioning. (Organization, 1992)

## Conclusions

Applying a matched cohort design to the Swedish population registries, death of close relatives was associated with, and likely contributed causally to, substantial increases in rates of SR, MD, and AUD, consistent with smaller prior clinical investigations. While our field has been able to ‘scale-up’ ascertainment of psychiatric disorders using national registries and large-scale biobanks, it has proven more difficult to obtain, in large representative population samples, high-quality measures of environmental adversities, especially those, like SLEs, occurring in adulthood in temporal proximity to disorder onset or recurrence. We tried to address this limitation by showing that we can accurately measure, in a large representative population, one class of severe SLEs – death of close relatives – which precede and likely cause substantial increases in rates of SR, MD, and AUD. Importantly, this approach eliminates the problem of biased reporting and selective recall which limits the interpretation of most prior SLE studies utilizing self-report. While individually often severe, however, death of relatives reflects, in most human populations, only a moderate proportion of the total adversities experienced in a lifetime. The next challenge is to extend this work to other objectively defined SLEs so that together they can be used, along with familial/genetic and other social/environmental risk factors, to develop, in high-quality registry samples, more realistic and comprehensive etiologic models for important psychiatric and substance-use disorders. In summary, our results suggest that it is feasible, in large representative samples, to integrate into risk disorder pathways that include genetic, social, and early developmental adversities, the major impact of exposures to selected environmental adversities of adulthood.

## Supporting information

Kendler et al. supplementary material Kendler et al. supplementary material

## Supplementary material

The supplementary material for this article can be found at https://doi.org/10.1017/S0033291723000570.
