# Supplementary material for: Death of parent, sibling, spouse, and child in a Swedish national sample and risk of subsequent stress reaction, major depression, alcohol-use disorder, and drug-use disorder
Source: Psychol Med. 2023 Apr 11;53(15):7138–50. doi: 10.1017/S0033291723000570 (PMC10719625; doi:10.1017/S0033291723000570)
Supplement: Kendler et al. supplementary material [file S0033291723000570sup001.docx]

Appendix for “Death of Parent, Sibling, Spouse and Child in a Swedish National Sample and Risk of Subsequent Stress Reaction, Major Depression, Alcohol Use Disorder and Drug Use Disorder”

Table 1

Details of the Data Set Used in These Analyses

Total Population Register, containing information about year of birth, sex, family and marital status; Multi-Generation Register, linking individuals born after 1932 to their parents; the Longitudinal Integration Database for Health Insurance and Labor Market Studies (LISA) containing information about education from 1990 to 2014; the Hospital Discharge Register, containing hospitalizations for Swedish inhabitants from 1964-2017; Prescribed Drug Register, containing all prescriptions in Sweden picked up by patients from July 2005 to 2017; Outpatient Care Register, containing information from all outpatient clinics from 2001 to 2017; the Mortality register, containing data and cause of death between 1952 to 2018, and regional Primary Health Care Registers including data from Blekinge (2009 - 2016), Dalarna (2005 - 2013), Värmland (2005 - 2015), Kalmar Län (2007 - 2016), Sörmland (1992 - 2017), Uppsala Län (2005 - 2015), Västernorrland (2008 - 2015) Norrbotten Län (2001 - 2014), Gävleborg (2010 - 2017), Gotland (2011 – 2018), Halland (2007 - 2014), Jönköpings Län (2008 - 2014), Kronoberg (2006 - 2016), Skåne (1989 - 2018), Västerbotten (1992- 2018), Östergötland (1990 - 2014), Stockholms Län (2003 - 2016), and Västra Götaland (2000 - 2013). The time-periods varies due to the regional differences in the timing of digitalizing of the patient records. In addition, we used for AUD and DUD, the Crime Register that included national complete data on all convictions in lower court from 1973-2017; Swedish Suspicion Register that included national data on individuals strongly suspected of crime from 1998-2015; and the Mortality Register with dates and causes of death from 1952 until 2016.

Table 2 Definitions of AUD and DUD

| Variable | Registers Used | Definition |
| --- | --- | --- |
| Alcohol Use Disorder (AUD) | The Swedish Hospital Discharge Register (coverage 1973-2014); Outpatient Care Register (national coverage 2001-2014); Primary Care Registry (Partly coverage from 1999-2014); the Swedish Drug Register (2005-2014); the Swedish Mortality Register, and the Swedish Criminal Register (1973-2014) and the Swedish Suspicion Register (1998-2014) | Alcohol Use Disorder (AUD) was identified in the Swedish medical and mortality registries by ICD -10 codes: E244, G312, G621, G721, I426, K292, K70, K852, K860, O354, T51, F10); in the Crime Register by codes 3005, 3201, which reflect crimes related to alcohol abuse; in the Suspicion Register by codes 0004, 0005 (Only those individuals with at least two alcohol-related crimes or suspicion of crimes from both Crime Register and Suspicion Register were included); in the Prescribed Drug Register by the drugs disulfiram (Anatomical Therapeutic Chemical (ATC) Classification System N07BB01), acamprosate (N07BB03), and naltrexone (N07BB04). |
| Drug Use Disorder (DUD) | The Swedish Hospital Discharge Register (coverage 1973-2014); Outpatient Care Register (national coverage 2001-2014); Primary Care Registry (Partly coverage from 1999-2014); the Swedish Drug Register (2005-2047); the Swedish Mortality Register, and the Swedish Criminal Register (1973-2014) and the Swedish Suspicion Register (1998-2014) | Drug abuse (DA) was identified in the Swedish medical and mortality registries by ICD-10: Mental and behavioral disorders due to psychoactive substance use (F10-F19), except those due to alcohol (F10) or tobacco (F17)); in the Suspicion Register by codes 3070, 5010, 5011, and 5012, that reflect crimes related to DA; and in the Crime Register by references to laws covering narcotics (law 1968:64, paragraph 1, point 6) and drug-related driving offences (law 1951:649, paragraph 4, subsection 2 and paragraph 4A, subsection 2). DA was identified in individuals (excluding those suffering from cancer) in the Prescribed Drug Register who had retrieved (in average) more than four defined daily doses a day for 12 months from either of Hypnotics and Sedatives (Anatomical Therapeutic Chemical (ATC) Classification System N05C and N05BA) or Opioids (ATC: N02A). |

Table 3

Matching Variables for Exposed and Unexposed Individuals*

|  | Death of | | | |
| --- | --- | --- | --- | --- |
| Matching Variables | Child | Spouse | Sibling | Parent |
| Sex, proband | Yes | Yes | Yes | Yes |
| Sex, relative | No | No | No | No |
| Birth year, proband | Yes (≤ 5 years) | Yes | Yes | Yes |
| Birth year, relative | Yes | Yes | Yes | No |
| Education | Yes | Yes | Yes | Yes |
| Marital status | No | Yes | No | No |
| *For rationale of limited matching, see(1) | | | | |

Table 4 – Model Formula

| $Y=\beta_{0}+ \beta_{1}t+\beta_{2}x_{exp}+ \beta_{3}x_{sex}+ \beta_{t5}{x_{exp}x}_{t5}+ \beta_{t6}{x_{exp}x}_{t6}+ \beta_{t7}x_{exp}x_{t7}+ \beta_{t8}x_{exp}x_{t8}+\beta_{t9}x_{exp}x_{t9}+ \beta_{t10}x_{exp}x_{t10}+ \beta_{t11}x_{exp}x_{t11}+ \beta_{t12}{x_{exp}x}_{t12}+ \beta_{t13}x_{exp}x_{t13}+\beta_{t14}{x_{exp}x}_{t14}+ \beta_{t15}x_{exp}x_{t15}+\beta_{t16}x_{exp}x_{t16}+ \beta_{13}tx_{sex}+ \beta_{23}x_{exp}x_{sex}+\beta_{3t5}{x_{exp}x}_{t5}x_{sex} + \beta_{3t6}{x_{exp}x}_{t6}x_{sex}+ \beta_{3t7}x_{exp}x_{t7}x_{sex}+ \beta_{3t8}x_{exp}x_{t8}x_{sex}+\beta_{3t9}x_{exp}x_{t9}x_{sex}+ \beta_{3t10}x_{exp}x_{t10}x_{sex}+ \beta_{3t11}x_{exp}x_{t11}x_{sex}+ \beta_{3t12}{x_{exp}x}_{t12}x_{sex}+ \beta_{3t13}x_{exp}x_{t13}x_{sex}+\beta_{3t14}{x_{exp}x}_{t14}x_{sex}+ \beta_{3t15}x_{exp}x_{t15}x_{sex}+\beta_{3t16}x_{exp}x_{t16}x_{sex}$ |
| --- |
| Where *t* is time, assessed as a continuous variable, *x_sex_* is 1 for men and 0 for women, *x_exp_* is 1 for exposed and 0 for unexposed controls. Further *x_t5_* is 1 for the 5^th^ assessed quarter, i.e., 10-12 months before the event, and 0 otherwise, *x_t6_* is 1 for the 6^th^ quarter, 7-9 months before and 0 otherwise, and so on, until *x_t16_* representing the period 22-24 months after the event. |

Table 5

Main Results of Association with Rates of Stress Response, Major Depression, Alcohol Use Disorder and Drug Use Disorder in the 2 years Before and After Death of a Parent, Sibling, Spouse and Child – Odds Ratios with 95% Confidence Intervals

| ***Death of parents*** | **Stress Response** | **Major Depression** | **Alcohol Use Disorder** | **Drug Use Disorder** |
| --- | --- | --- | --- | --- |
| ***Women*** | Expected difference between index group and controls, OR (95% CI) | | | |
| Index vs controls | 1.20 (1.17, 1.23) | 1.18 (1.16, 1.20) (#) | 1.11 (1.07, 1.16) | 1.21 (1.16, 1.26) |
|  | Additional risk increases in index group compared to controls, OR (95% CI) | | | |
| 22 to 24 months before | 1 | 1 | 1 | 1 |
| 19 to 21 months before | 1 | 1 | 1 | 1 |
| 16 to 19 months before | 1 | 1 | 1 | 1 |
| 13 to 16 months before | 1 | 1 | 1 | 1 |
| 10 to 13 months before | 1.08 (1.04, 1.12) | 1.02 (1.00, 1.05) | 1.04 (0.98, 1.12) | 1.03 (0.96, 1.10) |
| 7 to 10 months before | 1.11 (1.08, 1.15) | 1.03 (1.00, 1.06) | 1.04 (0.98, 1.12) | 1.07 (1.00, 1.15) |
| 4 to 7 months before | 1.18 (1.14, 1.22) | 1.06 (1.03, 1.09) | 1.06 (0.99, 1.13) | 1.11 (1.03, 1.19) |
| Up to 3 months before | 1.72 (1.67, 1.77) | 1.11 (1.08, 1.14) | 1.04 (0.97, 1.11) | 1.05 (0.97, 1.12) |
| Up to 3 monthe after | 4.70 (4.58, 4.82) | 1.30 (1.26, 1.33) | 1.07 (1.00, 1.15) | 1.06 (0.99, 1.14) |
| 4 to 6 months after | 1.63 (1.58, 1.69) | 1.25 (1.22, 1.29) | 1.12 (1.04, 1.20) | 1.07 (1.00, 1.16) |
| 7 to 9 months after | 1.35 (1.31, 1.40) | 1.20 (1.17, 1.23) | 1.09 (1.01, 1.17) | 1.08 (1.00, 1.16) |
| 10 to 12 months after | 1.22 (1.18, 1.27) | 1.17 (1.13, 1.20) | 1.05 (0.98, 1.13) | 1.01 (0.94, 1.10) |
| 13 to 15 months after | 1.14 (1.10, 1.19) | 1.15 (1.12, 1.18) | 1.08 (1.01, 1.16) | 1.04 (0.96, 1.13) |
| 16 to 18 months after | 1.08 (1.04, 1.13) | 1.09 (1.06, 1.13) | 1.07 (0.99, 1.15) | 1.04 (0.96, 1.13) |
| 19 to 21 months after | 1.06 (1.02, 1.10) | 1.07 (1.04, 1.11) | 1.13 (1.05, 1.22) | 1.00 (0.92, 1.09) |
| 22 to 24 months after | 1.01 (0.97, 1.05) | 1.06 (1.02, 1.09) | 1.05 (0.97, 1.13) | 1.03 (0.95, 1.13) |
|  |  |  | | |
| *Men* | Expected difference between index group and controls, OR (95% CI) | | | |
| Index vs controls | 1.16 (1.12, 1.20) | 1.10 (1.08, 1.13) | 1.08 (1.05, 1.10) | 1.24 (1.21, 1.28) |
|  | Additional risk increase in index group compared to controls, OR (95% CI) | | | |
| 22 to 24 months before | 1 | 1 | 1 | 1 |
| 19 to 21 months before | 1 | 1 | 1 | 1 |
| 16 to 19 months before | 1 | 1 | 1 | 1 |
| 13 to 16 months before | 1 | 1 | 1 | 1 |
| 10 to 13 months before | 1.02 (0.96, 1.09) | 1.06 (1.02, 1.10) | 1.05 (1.01, 1.09) | 1.01 (0.97, 1.06) |
| 7 to 10 months before | 1.08 (1.02, 1.15) | 1.08 (1.03, 1.12) | 1.01 (0.96, 1.05) | 1.02 (0.97, 1.07) |
| 4 to 7 months before | 1.09 (1.02, 1.15) | 1.05 (1.01, 1.10) | 1.05 (1.01, 1.10) | 1.04 (0.99, 1.09) |
| Up to 3 months before | 1.53 (1.45, 1.61) (#) | 1.08 (1.04, 1.13) | 1.07 (1.03, 1.12) | 1.05 (1.00, 1.10) |
| Up to 3 months after | 4.52 (4.34, 4.71) | 1.27 (1.23, 1.32) | 1.08 (1.04, 1.13) | 1.04 (1.00, 1.10) |
| 4 to 6 months after | 1.59 (1.51, 1.68) | 1.22 (1.18, 1.27) | 1.09 (1.04, 1.14) | 1.07 (1.02, 1.12) |
| 7 to 9 months after | 1.33 (1.25, 1.41) | 1.22 (1.17, 1.26) | 1.10 (1.06, 1.15) | 1.07 (1.02, 1.12) |
| 10 to 12 months after | 1.20 (1.13, 1.28) | 1.19 (1.15, 1.24) | 1.09 (1.05, 1.14) | 1.01 (0.96, 1.06) |
| 13 to 15 months after | 1.14 (1.07, 1.22) | 1.16 (1.11, 1.21) | 1.08 (1.03, 1.13) | 1.02 (0.96, 1.07) |
| 16 to 18 months after | 1.08 (1.02, 1.16) | 1.13 (1.08, 1.17) | 1.08 (1.03, 1.13) | 1.02 (0.97, 1.08) |
| 19 to 21 months after | 1.07 (1.00, 1.15) | 1.12 (1.07, 1.16) | 1.09 (1.04, 1.14) | 1.01 (0.95, 1.06) |
| 22 to 24 months after | 1.05 (0.98, 1.13) | 1.11 (1.06, 1.16) | 1.06 (1.01, 1.11) | 0.99 (0.94, 1.05) |
|  |  |  |  |  |
| ***Death of Siblings*** |  |  |  |  |
| *Women* | Expected difference between index group and controls, OR (95% CI) | | | |
| Index vs controls | 1.18 (1.13, 1.24) | 1.26 (1.23, 1.3) | 1.51 (1.41, 1.62) | 1.86 (1.70, 2.04) |
|  | Additional risk increase in index group compared to controls, OR (95% CI) | | | |
| 22 to 24 months before | 1 | 1 | 1 | 1 |
| 19 to 21 months before | 1 | 1 | 1 | 1 |
| 16 to 19 months before | 1 | 1 | 1 | 1 |
| 13 to 16 months before | 1 | 1 | 1 | 1 |
| 10 to 13 months before | 1.04 (0.96, 1.13) | 1.05 (1.00, 1.11) | 1.02 (0.91, 1.14) | 1.04 (0.91, 1.20) |
| 7 to 10 months before | 1.04 (0.96, 1.13) | 1.03 (0.98, 1.09) | 1.10 (0.99, 1.23) | 1.11 (0.97, 1.28) |
| 4 to 7 months before | 1.13 (1.04, 1.22) | 1.05 (1.00, 1.10) | 1.00 (0.89, 1.12) | 1.26 (1.10, 1.44) |
| Up to 3 months before | 1.54 (1.43, 1.65) | 1.09 (1.04, 1.15) | 1.05 (0.93, 1.17) | 1.17 (1.02, 1.34) |
| Up to 3 months after | 5.16 (4.89, 5.45) | 1.30 (1.23, 1.36) | 1.12 (1.00, 1.25) | 1.08 (0.94, 1.25) |
| 4 to 6 months after | 1.80 (1.68, 1.93) | 1.25 (1.19, 1.32) | 1.16 (1.04, 1.30) | 1.13 (0.98, 1.31) |
| 7 to 9 months after | 1.45 (1.34, 1.56) | 1.22 (1.16, 1.29) | 1.10 (0.97, 1.24) | 1.17 (1.01, 1.36) |
| 10 to 12 months after | 1.31 (1.21, 1.42) | 1.23 (1.16, 1.29) | 1.15 (1.02, 1.30) | 1.03 (0.88, 1.21) |
| 13 to 15 months after | 1.18 (1.08, 1.28) | 1.18 (1.11, 1.24) | 1.09 (0.96, 1.24) | 0.98 (0.83, 1.16) |
| 16 to 18 months after | 1.12 (1.02, 1.22) | 1.10 (1.04, 1.17) | 1.09 (0.96, 1.24) | 1.04 (0.88, 1.23) |
| 19 to 21 months after | 1.07 (0.97, 1.17) | 1.08 (1.02, 1.15) | 1.09 (0.95, 1.25) | 1.05 (0.88, 1.25) |
| 22 to 24 months after | 1.01 (0.92, 1.11) | 1.06 (0.99, 1.12) | 1.08 (0.94, 1.24) | 1.02 (0.85, 1.23) |
|  |  |  | | |
| *Men* | Expected difference between index group and controls, OR (95% CI) | | | |
| Index vs controls | 1.33 (1.23, 1.44) | 1.30 (1.25, 1.36) | 1.51 (1.45, 1.57) | 2.10 (1.97, 2.24) |
|  | Additional risk increase in index group compared to controls, OR (95% CI) | | | |
| 22 to 24 months before | 1 | 1 | 1 | 1 |
| 19 to 21 months before | 1 | 1 | 1 | 1 |
| 16 to 19 months before | 1 | 1 | 1 | 1 |
| 13 to 16 months before | 1 | 1 | 1 | 1 |
| 10 to 13 months before | 1.00 (0.88, 1.14) | 1.06 (0.99, 1.14) | 1.05 (0.99, 1.12) | 1.02 (0.93, 1.12) |
| 7 to 10 months before | 0.99 (0.86, 1.12) | 1.05 (0.98, 1.12) | 1.04 (0.98, 1.11) | 1.07 (0.97, 1.18) |
| 4 to 7 months before | 0.98 (0.86, 1.12) | 1.07 (1.00, 1.15) | 1.10 (1.04, 1.18) | 1.09 (0.99, 1.20) |
| Up to 3 months before | 1.26 (1.12, 1.43) (*) | 1.06 (0.99, 1.14) | 1.10 (1.03, 1.18) | 1.08 (0.97, 1.19) |
| Up to 3 months after | 5.35 (4.89, 5.84) | 1.27 (1.19, 1.36) | 1.24 (1.17, 1.32) | 1.21 (1.09, 1.33) |
| 4 to 6 months after | 1.68 (1.50, 1.89) | 1.28 (1.20, 1.37) | 1.22 (1.14, 1.30) | 1.08 (0.98, 1.20) |
| 7 to 9 months after | 1.31 (1.15, 1.49) | 1.21 (1.12, 1.30) | 1.20 (1.12, 1.28) | 1.16 (1.04, 1.29) |
| 10 to 12 months after | 1.14 (0.99, 1.30) | 1.18 (1.09, 1.27) | 1.15 (1.08, 1.24) | 1.03 (0.92, 1.16) |
| 13 to 15 months after | 1.07 (0.92, 1.23) | 1.11 (1.03, 1.20) | 1.14 (1.06, 1.23) | 1.11 (0.99, 1.24) |
| 16 to 18 months after | 1.08 (0.93, 1.25) | 1.16 (1.07, 1.25) | 1.16 (1.08, 1.25) | 1.05 (0.93, 1.19) |
| 19 to 21 months after | 0.92 (0.79, 1.07) | 1.09 (1.01, 1.18) | 1.20 (1.11, 1.29) | 1.01 (0.88, 1.14) |
| 22 to 24 months after | 1.05 (0.90, 1.22) | 1.08 (0.99, 1.17) | 1.15 (1.06, 1.24) | 1.04 (0.91, 1.19) |
|  |  |  |  |  |
| ***Death of Spouse*** |  |  |  |  |
| *Women* | Expected difference between index group and controls, OR (95% CI) | | | |
| Index vs controls | 2.36 (2.23, 2.50) | 1.44 (1.40, 1.48) | 1.72 (1.57, 1.89) | 1.50 (1.32, 1.71) |
|  | Additional risk increase in index group compared to controls, OR (95% CI) | | | |
| 22 to 24 months before | 1 | 1 | 1 | 1 |
| 19 to 21 months before | 1 | 1 | 1 | 1 |
| 16 to 19 months before | 1 | 1 | 1 | 1 |
| 13 to 16 months before | 1 | 1 | 1 | 1 |
| 10 to 13 months before | 1.35 (1.26, 1.45) | 1.10 (1.06, 1.15) | 1.10 (0.96, 1.27) | 1.05 (0.86, 1.28) |
| 7 to 10 months before | 1.51 (1.41, 1.62) | 1.14 (1.09, 1.19) | 1.22 (1.07, 1.40) | 1.10 (0.90, 1.34) |
| 4 to 7 months before | 1.89 (1.77, 2.02) | 1.23 (1.18, 1.28) | 1.17 (1.02, 1.35) | 1.13 (0.93, 1.38) |
| Up to 3 months before | 3.7 (3.49, 3.92) | 1.41 (1.35, 1.46) | 1.24 (1.08, 1.42) | 1.16 (0.95, 1.42) |
| Up to 3 monthe after | 16.64 (15.79, 17.54) | 2.16 (2.08, 2.24) | 1.78 (1.57, 2.02) | 1.40 (1.16, 1.70) |
| 4 to 6 months after | 4.85 (4.56, 5.16) | 2.38 (2.29, 2.47) | 1.71 (1.49, 1.95) | 1.28 (1.05, 1.56) |
| 7 to 9 months after | 2.75 (2.57, 2.95) | 2.15 (2.06, 2.23) | 1.58 (1.37, 1.82) | 1.16 (0.94, 1.43) |
| 10 to 12 months after | 1.82 (1.69, 1.97) | 1.99 (1.91, 2.07) | 1.54 (1.33, 1.78) | 0.99 (0.79, 1.24) |
| 13 to 15 months after | 1.28 (1.17, 1.39) | 1.82 (1.75, 1.90) | 1.49 (1.28, 1.73) | 1.12 (0.90, 1.40) |
| 16 to 18 months after | 0.93 (0.85, 1.03) | 1.66 (1.59, 1.74) | 1.30 (1.10, 1.53) | 0.85 (0.67, 1.09) |
| 19 to 21 months after | 0.80 (0.73, 0.89) | 1.55 (1.48, 1.62) | 1.32 (1.11, 1.56) | 0.94 (0.73, 1.20) |
| 22 to 24 months after | 0.66 (0.59, 0.73) | 1.49 (1.41, 1.56) | 1.32 (1.11, 1.57) | 0.88 (0.68, 1.14) |
|  |  |  |  |  |
| *Men* | Expected difference between index group and controls, OR (95% CI) | | | |
| Index vs controls | 2.86 (2.51, 3.26 (*) | 1.22 (1.16, 1.29) (#) | 1.72 (1.57, 1.88) | 1.81 (1.48, 2.21) |
|  | Additional risk increases in index group compared to controls, OR (95% CI) | | | |
| 22 to 24 months before | 1 | 1 | 1 | 1 |
| 19 to 21 months before | 1 | 1 | 1 | 1 |
| 16 to 19 months before | 1 | 1 | 1 | 1 |
| 13 to 16 months before | 1 | 1 | 1 | 1 |
| 10 to 13 months before | 1.24 (1.06, 1.46) | 1.11 (1.02, 1.20) | 1.04 (0.90, 1.19) | 1.02 (0.75, 1.37) |
| 7 to 10 months before | 1.61 (1.39, 1.87) | 1.22 (1.12, 1.32) | 1.01 (0.88, 1.17) | 1.13 (0.84, 1.51) |
| 4 to 7 months before | 2.15 (1.87, 2.47) | 1.34 (1.23, 1.45) | 1.13 (0.99, 1.30) | 1.11 (0.82, 1.49) |
| Up to 3 months before | 4.95 (4.37, 5.6) (#) | 1.66 (1.53, 1.79) (#) | 1.35 (1.18, 1.54) | 1.40 (1.06, 1.85) |
| Up to 3 months after | 22.9 (20.33, 25.79) (#) | 2.95 (2.76, 3.15) (#) | 2.39 (2.13, 2.68) (#) | 1.48 (1.12, 1.96) |
| 4 to 6 months after | 5.28 (4.59, 6.07) | 2.95 (2.75, 3.16) (#) | 2.2 (1.95, 2.48) (*) | 1.36 (1.01, 1.82) |
| 7 to 9 months after | 3.03 (2.59, 3.54) | 2.64 (2.46, 2.84) (#) | 2.22 (1.96, 2.52) (#) | 1.22 (0.89, 1.66) |
| 10 to 12 months after | 1.90 (1.59, 2.26) | 2.33 (2.16, 2.51) (#) | 1.92 (1.68, 2.20) | 1.24 (0.91, 1.71) |
| 13 to 15 months after | 1.28 (1.05, 1.56) | 2.18 (2.01, 2.36) (#) | 2.00 (1.74, 2.29) (*) | 1.03 (0.73, 1.45) |
| 16 to 18 months after | 0.89 (0.71, 1.11) | 1.90 (1.75, 2.07) (*) | 1.89 (1.64, 2.19) (#) | 0.99 (0.70, 1.42) |
| 19 to 21 months after | 0.69 (0.54, 0.88) | 1.77 (1.62, 1.94) (*) | 1.74 (1.50, 2.03) | 0.81 (0.55, 1.18) |
| 22 to 24 months after | 0.64 (0.50, 0.83) | 1.63 (1.49, 1.79) | 1.79 (1.53, 2.10) | 0.96 (0.66, 1.40) |
|  |  |  |  |  |
| ***Death of Child*** |  |  |  |  |
| *Women* | Expected difference between index group and controls, OR (95% CI) | | | |
| Index vs controls | 1.99 (1.65, 2.41) | 1.51 (1.27, 1.79) |  |  |
|  | Additional risk increases in index group compared to controls, OR (95% CI) | | | |
| 22 to 24 months before | 1 | 1 |  |  |
| 19 to 21 months before | 1 | 1 |  |  |
| 16 to 19 months before | 1 | 1 |  |  |
| 13 to 16 months before | 1 | 1 |  |  |
| 10 to 13 months before | 1.25 (0.96, 1.61) | 1.06 (0.81, 1.37) |  |  |
| 7 to 10 months before | 1.12 (0.86, 1.46) | 0.75 (0.56, 1.01) |  |  |
| 4 to 7 months before | 1.16 (0.89, 1.51) | 0.92 (0.70, 1.21) |  |  |
| Up to 3 months before | 2.43 (1.95, 3.03) | 1.21 (0.94, 1.55) |  |  |
| Up to 3 monthe after | 31.3 (26.17, 37.43) | 4.45 (3.68, 5.38) |  |  |
| 4 to 6 months after | 13.17 (10.83, 16.01) | 5.27 (4.35, 6.38) |  |  |
| 7 to 9 months after | 7.03 (5.67, 8.72) | 4.31 (3.51, 5.28) |  |  |
| 10 to 12 months after | 5.29 (4.2, 6.67) | 3.45 (2.77, 4.29) |  |  |
| 13 to 15 months after | 3.79 (2.95, 4.86) | 2.68 (2.12, 3.39) |  |  |
| 16 to 18 months after | 2.67 (2.03, 3.51) | 2.39 (1.86, 3.06) |  |  |
| 19 to 21 months after | 2.17 (1.62, 2.9) | 2.19 (1.68, 2.84) |  |  |
| 22 to 24 months after | 1.9 (1.39, 2.58) | 1.81 (1.37, 2.39) |  |  |
|  |  |  |  |  |
| *Men* | Expected difference between index group and controls, OR (95% CI) | | | |
| Index vs controls | 1.85 (1.4, 2.45) | 1.73 (1.36, 2.2) |  |  |
|  | Additional risk increases in index group compared to controls, OR (95% CI) | | | |
| 22 to 24 months before | 1 | 1 |  |  |
| 19 to 21 months before | 1 | 1 |  |  |
| 16 to 19 months before | 1 | 1 |  |  |
| 13 to 16 months before | 1 | 1 |  |  |
| 10 to 13 months before | 1.09 (0.72, 1.65) | 0.94 (0.65, 1.36) |  |  |
| 7 to 10 months before | 1.34 (0.91, 1.98) | 1.05 (0.74, 1.49) |  |  |
| 4 to 7 months before | 1.23 (0.82, 1.84) | 0.81 (0.55, 1.2) |  |  |
| Up to 3 months before | 2.28 (1.61, 3.22) | 1.1 (0.78, 1.56) |  |  |
| Up to 3 monthe after | 42.26 (32.37, 55.16) | 4.51 (3.49, 5.82) |  |  |
| 4 to 6 months after | 13.15 (9.77, 17.7) | 3.83 (2.92, 5.03) |  |  |
| 7 to 9 months after | 8.17 (5.9, 11.32) | 2.97 (2.22, 3.99) |  |  |
| 10 to 12 months after | 6.25 (4.39, 8.9) | 2.77 (2.04, 3.77) |  |  |
| 13 to 15 months after | 4.6 (3.14, 6.75) | 2.21 (1.59, 3.08) |  |  |
| 16 to 18 months after | 3.13 (2.06, 4.76) | 1.98 (1.4, 2.8) |  |  |
| 19 to 21 months after | 2.72 (1.74, 4.26) | 1.77 (1.23, 2.55) |  |  |
| 22 to 24 months after | 2.64 (1.65, 4.23) | 1.5 (1.02, 2.2) |  |  |
| Odds ratios are all set to unity for the first 4 observation periods which were used to predict the results for case subjects had they not experienced the death of a close relative. To indicate significant differences between males and females I have added, in the columns for females, (*) representing p<0.01 and (#) representing p<0.001 | | | | |

Table 6

Comparison of the Association with Rates of Stress Response, Major Depression, Alcohol Use Disorder and Drug Use Disorder in the 2 years Before and After Death of a Parent, Sibling, Spouse When the Deceased Relative was < 65 Years of Age versus ≥ 65 Years of Age and when the Death of a Child was versus was not Accidental – Odds Ratios with 95% Confidence Intervals

| ***Death of parents*** | **Stress Response** | **Major Depression** | **Alcohol Use Disorder** | **Drug Use Disorder** |
| --- | --- | --- | --- | --- |
| *Death at or before age 65* |  | |  | |
| Index vs controls | 1.42 (1.35, 1.48) | 1.25 (1.21, 1.29) | 1.54 (1.45, 1.64) | 1.61 (1.54, 1.67) |
|  | Additional risk increases in index group compared to controls, OR (95% CI) | | | |
| 22 to 24 months before | 1 | 1 | 1 | 1 |
| 19 to 21 months before | 1 | 1 | 1 | 1 |
| 16 to 19 months before | 1 | 1 | 1 | 1 |
| 13 to 16 months before | 1 | 1 | 1 | 1 |
| 10 to 13 months before | 1.10 (1.03, 1.19) | 1.05 (1.00, 1.11) | 1.01 (0.91, 1.11) | 1.04 (0.98, 1.10) |
| 7 to 10 months before | 1.13 (1.05, 1.21) | 1.05 (1.00, 1.11) | 0.99 (0.89, 1.09) | 1.06 (0.99, 1.13) |
| 4 to 7 months before | 1.30 (1.22, 1.39) | 1.09 (1.04, 1.15) | 1.14 (1.04, 1.26) | 1.07 (1.01, 1.14) |
| Up to 3 months before | 2.31 (2.18, 2.45) | 1.14 (1.08, 1.20) | 1.13 (1.02, 1.24) | 1.03 (0.97, 1.10) |
| Up to 3 months after | 9.26 (8.83, 9.70) | 1.55 (1.47, 1.62) | 1.18 (1.07, 1.3) | 1.08 (1.01, 1.15) |
| 4 to 6 months after | 2.33 (2.20, 2.48) | 1.43 (1.37, 1.51) | 1.22 (1.11, 1.35) | 1.06 (0.99, 1.13) |
| 7 to 9 months after | 1.71 (1.60, 1.82) | 1.38 (1.31, 1.45) | 1.15 (1.04, 1.27) | 1.09 (1.02, 1.16) |
| 10 to 12 months after | 1.42 (1.33, 1.53) | 1.33 (1.26, 1.40) | 1.11 (1.00, 1.24) | 1.04 (0.97, 1.11) |
| 13 to 15 months after | 1.26 (1.17, 1.36) | 1.26 (1.19, 1.33) | 1.09 (0.98, 1.21) | 1.03 (0.96, 1.10) |
| 16 to 18 months after | 1.12 (1.04, 1.21) | 1.20 (1.14, 1.27) | 1.04 (0.93, 1.16) | 1.01 (0.94, 1.09) |
| 19 to 21 months after | 1.07 (0.99, 1.16) | 1.15 (1.09, 1.22) | 1.19 (1.06, 1.33) | 1.02 (0.95, 1.10) |
| 22 to 24 months after | 1.01 (0.93, 1.10) | 1.15 (1.09, 1.22) | 1.17 (1.04, 1.31) | 1.00 (0.92, 1.08) |
|  |  | |  | |
| *Death after age 65* | Expected difference between index group and controls, OR (95% CI) | | | |
| Index vs controls | 1.15 (1.13, 1.17) (#) | 1.13 (1.12, 1.15) (#) | 1.04 (1.02, 1.06) (#) | 1.09 (1.06, 1.12) (#) |
|  | Additional risk increases in index group compared to controls, OR (95% CI) | | | |
| 22 to 24 months before | 1 | 1 | 1 | 1 |
| 19 to 21 months before | 1 | 1 | 1 | 1 |
| 16 to 19 months before | 1 | 1 | 1 | 1 |
| 13 to 16 months before | 1 | 1 | 1 | 1 |
| 10 to 13 months before | 1.06 (1.02, 1.09) | 1.03 (1.00, 1.06) | 1.05 (1.01, 1.09) | 1.00 (0.95, 1.05) |
| 7 to 10 months before | 1.10 (1.06, 1.14) | 1.04 (1.01, 1.07) | 1.02 (0.98, 1.06) | 1.00 (0.96, 1.06) |
| 4 to 7 months before | 1.12 (1.08, 1.16) (#) | 1.05 (1.02, 1.07) | 1.04 (1.00, 1.08) | 1.04 (0.99, 1.09) |
| Up to 3 months before | 1.52 (1.47, 1.56) (#) | 1.09 (1.07, 1.12) | 1.05 (1.01, 1.09) | 1.04 (0.99, 1.09) |
| Up to 3 months after | 3.59 (3.50, 3.68) (#) | 1.23 (1.20, 1.26) (#) | 1.06 (1.02, 1.10) | 1.01 (0.96, 1.06) |
| 4 to 6 months after | 1.46 (1.42, 1.51) (#) | 1.2 (1.17, 1.23) (#) | 1.07 (1.03, 1.11) | 1.07 (1.01, 1.12) |
| 7 to 9 months after | 1.27 (1.23, 1.31) (#) | 1.16 (1.13, 1.19) (#) | 1.09 (1.05, 1.13) | 1.04 (0.98, 1.09) |
| 10 to 12 months after | 1.18 (1.14, 1.22) (#) | 1.14 (1.11, 1.17) (#) | 1.07 (1.03, 1.12) | 0.98 (0.93, 1.04) |
| 13 to 15 months after | 1.12 (1.08, 1.16) (*) | 1.13 (1.10, 1.16) (#) | 1.08 (1.03, 1.12) | 1.01 (0.95, 1.07) |
| 16 to 18 months after | 1.08 (1.04, 1.12) | 1.08 (1.05, 1.11) (#) | 1.08 (1.03, 1.12) | 1.01 (0.96, 1.07) |
| 19 to 21 months after | 1.06 (1.02, 1.10) | 1.07 (1.04, 1.10) | 1.08 (1.04, 1.13) | 0.98 (0.93, 1.04) |
| 22 to 24 months after | 1.02 (0.98, 1.06) | 1.05 (1.02, 1.09) (*) | 1.03 (0.99, 1.08) | 0.99 (0.94, 1.05) |
|  |  |  |  |  |
| ***Death of Siblings*** |  |  |  |  |
| *Death at or before age 65* | Expected difference between index group and controls, OR (95% CI) | | | |
| Index vs controls | 1.23 (1.17, 1.29) | 1.29 (1.25, 1.34) | 1.69 (1.62, 1.77) | 2.11 (1.99, 2.24) |
|  | Additional risk increases in index group compared to controls, OR (95% CI) | | | |
| 22 to 24 months before | 1 | 1 | 1 | 1 |
| 19 to 21 months before | 1 | 1 | 1 | 1 |
| 16 to 19 months before | 1 | 1 | 1 | 1 |
| 13 to 16 months before | 1 | 1 | 1 | 1 |
| 10 to 13 months before | 1.03 (0.95, 1.12) | 1.03 (0.97, 1.09) | 1.06 (0.99, 1.13) | 1.04 (0.95, 1.13) |
| 7 to 10 months before | 1.05 (0.97, 1.14) | 1.01 (0.95, 1.07) | 1.01 (0.94, 1.09) | 1.08 (0.99, 1.17) |
| 4 to 7 months before | 1.14 (1.05, 1.23) | 1.04 (0.98, 1.10) | 1.06 (0.99, 1.14) | 1.13 (1.04, 1.23) |
| Up to 3 months before | 1.54 (1.44, 1.66) | 1.06 (1.01, 1.13) | 1.05 (0.98, 1.13) | 1.09 (1.00, 1.20) |
| Up to 3 months after | 6.37 (6.03, 6.72) | 1.38 (1.31, 1.45) | 1.15 (1.07, 1.24) | 1.18 (1.08, 1.29) |
| 4 to 6 months after | 1.98 (1.84, 2.12) | 1.31 (1.24, 1.38) | 1.14 (1.06, 1.22) | 1.10 (1.00, 1.21) |
| 7 to 9 months after | 1.52 (1.41, 1.64) | 1.28 (1.21, 1.35) | 1.12 (1.04, 1.20) | 1.16 (1.06, 1.28) |
| 10 to 12 months after | 1.32 (1.22, 1.43) | 1.22 (1.15, 1.29) | 1.10 (1.02, 1.19) | 1.02 (0.92, 1.13) |
| 13 to 15 months after | 1.18 (1.09, 1.29) | 1.17 (1.11, 1.25) | 1.04 (0.96, 1.13) | 1.08 (0.97, 1.20) |
| 16 to 18 months after | 1.15 (1.06, 1.26) | 1.14 (1.07, 1.21) | 1.04 (0.96, 1.13) | 1.08 (0.97, 1.21) |
| 19 to 21 months after | 1.01 (0.92, 1.10) | 1.10 (1.03, 1.17) | 1.07 (0.98, 1.16) | 1.01 (0.90, 1.13) |
| 22 to 24 months after | 1.01 (0.92, 1.11) | 1.05 (0.98, 1.12) | 1.06 (0.97, 1.15) | 1.04 (0.92, 1.17) |
|  |  | |  | |
| *Death after age 65* | Expected difference between index group and controls, OR (95% CI) | | | |
| Index vs controls | 1.20 (1.11, 1.28) | 1.26 (1.21, 1.30) | 1.29 (1.22, 1.36) (#) | 1.68 (1.49, 1.89) (#) |
|  | Additional risk increases in index group compared to controls, OR (95% CI) | | | |
| 22 to 24 months before | 1 | 1 | 1 | 1 |
| 19 to 21 months before | 1 | 1 | 1 | 1 |
| 16 to 19 months before | 1 | 1 | 1 | 1 |
| 13 to 16 months before | 1 | 1 | 1 | 1 |
| 10 to 13 months before | 1.03 (0.91, 1.16) | 1.09 (1.02, 1.15) | 1.01 (0.93, 1.11) | 0.97 (0.8, 1.17) |
| 7 to 10 months before | 0.99 (0.88, 1.12) | 1.08 (1.02, 1.15) | 1.12 (1.03, 1.22) | 1.11 (0.92, 1.33) |
| 4 to 7 months before | 0.97 (0.86, 1.10) | 1.08 (1.01, 1.15) | 1.10 (1.00, 1.20) | 1.2 (1, 1.44) |
| Up to 3 months before | 1.29 (1.14, 1.45) (*) | 1.11 (1.04, 1.18) | 1.14 (1.04, 1.25) | 1.15 (0.95, 1.39) |
| Up to 3 months after | 2.43 (2.20, 2.68) (#) | 1.17 (1.10, 1.25) (#) | 1.29 (1.18, 1.42) | 1.07 (0.88, 1.3) |
| 4 to 6 months after | 1.25 (1.10, 1.41) (#) | 1.20 (1.13, 1.28) | 1.30 (1.19, 1.42) | 1.06 (0.87, 1.3) |
| 7 to 9 months after | 1.13 (0.99, 1.29) (#) | 1.14 (1.07, 1.21) (*) | 1.24 (1.13, 1.37) | 1.14 (0.93, 1.4) |
| 10 to 12 months after | 1.09 (0.96, 1.25) | 1.19 (1.12, 1.27) | 1.22 (1.10, 1.34) | 1.07 (0.86, 1.32) |
| 13 to 15 months after | 1.04 (0.90, 1.20) | 1.12 (1.05, 1.20) | 1.26 (1.14, 1.39) (*) | 0.95 (0.75, 1.19) |
| 16 to 18 months after | 0.96 (0.83, 1.12) | 1.09 (1.02, 1.17) | 1.28 (1.16, 1.42) (*) | 0.85 (0.66, 1.08) |
| 19 to 21 months after | 1.07 (0.93, 1.25) | 1.06 (0.98, 1.14) | 1.32 (1.19, 1.47) (*) | 1.06 (0.84, 1.35) |
| 22 to 24 months after | 1.04 (0.89, 1.21) | 1.08 (1.01, 1.17) | 1.22 (1.09, 1.36) | 0.98 (0.77, 1.26) |
|  |  |  |  |  |
| ***Death of Spouse*** |  |  |  |  |
| *Death at or before age 65* | Expected difference between index group and controls, OR (95% CI) | | | |
| Index vs controls | 2.62 (2.42, 2.84) | 1.41 (1.32, 1.50) | 1.83 (1.62, 2.07) | 2.65 (2.16, 3.25) |
|  | Additional risk increases in index group compared to controls, OR (95% CI) | | | |
| 22 to 24 months before | 1 | 1 | 1 | 1 |
| 19 to 21 months before | 1 | 1 | 1 | 1 |
| 16 to 19 months before | 1 | 1 | 1 | 1 |
| 13 to 16 months before | 1 | 1 | 1 | 1 |
| 10 to 13 months before | 1.47 (1.33, 1.61) | 1.1 (1.00, 1.22) | 1.14 (0.96, 1.35) | 1.09 (0.83, 1.42) |
| 7 to 10 months before | 1.75 (1.60, 1.91) | 1.23 (1.12, 1.36) | 1.07 (0.89, 1.28) | 1.10 (0.84, 1.43) |
| 4 to 7 months before | 2.25 (2.06, 2.45) | 1.49 (1.35, 1.63) | 1.12 (0.94, 1.33) | 0.82 (0.61, 1.11) |
| Up to 3 months before | 4.06 (3.75, 4.39) | 1.96 (1.80, 2.14) | 1.26 (1.06, 1.50) | 1.23 (0.95, 1.61) |
| Up to 3 months after | 18.01 (16.71, 19.40) | 3.61 (3.34, 3.90) | 2.34 (2.02, 2.71) | 1.35 (1.04, 1.76) |
| 4 to 6 months after | 4.48 (4.11, 4.89) | 3.37 (3.10, 3.65) | 2.14 (1.83, 2.50) | 1.16 (0.87, 1.53) |
| 7 to 9 months after | 2.73 (2.47, 3.01) | 2.96 (2.71, 3.23) | 2.12 (1.80, 2.49) | 0.94 (0.69, 1.28) |
| 10 to 12 months after | 1.74 (1.56, 1.95) | 2.59 (2.36, 2.84) | 1.98 (1.67, 2.35) | 0.93 (0.67, 1.27) |
| 13 to 15 months after | 1.32 (1.17, 1.49) | 2.39 (2.17, 2.63) | 1.97 (1.65, 2.35) | 1.03 (0.75, 1.41) |
| 16 to 18 months after | 0.96 (0.84, 1.09) | 2.11 (1.90, 2.33) | 1.84 (1.53, 2.21) | 0.76 (0.53, 1.08) |
| 19 to 21 months after | 0.81 (0.70, 0.93) | 1.91 (1.72, 2.13) | 1.64 (1.35, 1.99) | 0.77 (0.53, 1.10) |
| 22 to 24 months after | 0.75 (0.65, 0.87) | 1.87 (1.67, 2.09) | 1.62 (1.32, 1.98) | 0.73 (0.50, 1.07) |
|  |  |  |  |  |
| *Males* | Expected difference between index group and controls, OR (95% CI) | | | |
| Index vs controls | 2.31 (2.16, 2.47) | 1.39 (1.35, 1.43) | 1.68 (1.55, 1.81) | 1.29 (1.13, 1.46) (#) |
| *Death after age 65* | Additional risk increases in index group compared to controls, OR (95% CI) | | | |
| 22 to 24 months before | 1 | 1 | 1 | 1 |
| 19 to 21 months before | 1 | 1 | 1 | 1 |
| 16 to 19 months before | 1 | 1 | 1 | 1 |
| 13 to 16 months before | 1 | 1 | 1 | 1 |
| 10 to 13 months before | 1.23 (1.13, 1.34) (*) | 1.10 (1.06, 1.15) | 1.04 (0.92, 1.17) | 1.00 (0.81, 1.24) |
| 7 to 10 months before | 1.37 (1.26, 1.49) (#) | 1.14 (1.09, 1.19) | 1.14 (1.01, 1.28) | 1.10 (0.89, 1.35) |
| 4 to 7 months before | 1.71 (1.57, 1.85) (#) | 1.21 (1.16, 1.26) (#) | 1.17 (1.04, 1.31) | 1.29 (1.06, 1.57) |
| Up to 3 months before | 3.86 (3.60, 4.14) | 1.37 (1.32, 1.43) (#) | 1.31 (1.17, 1.47) | 1.21 (0.98, 1.48) |
| Up to 3 months after | 18.63 (17.49, 19.84) | 2.11 (2.03, 2.18) (#) | 1.98 (1.78, 2.19) | 1.43 (1.17, 1.74) |
| 4 to 6 months after | 5.33 (4.95, 5.74) (*) | 2.35 (2.27, 2.44) (#) | 1.87 (1.68, 2.09) | 1.35 (1.10, 1.66) |
| 7 to 9 months after | 2.88 (2.65, 3.13) | 2.13 (2.05, 2.21) (#) | 1.80 (1.60, 2.02) | 1.28 (1.04, 1.59) |
| 10 to 12 months after | 1.91 (1.74, 2.10) | 1.97 (1.89, 2.04) (#) | 1.61 (1.42, 1.82) | 1.12 (0.89, 1.40) |
| 13 to 15 months after | 1.24 (1.12, 1.38) | 1.81 (1.74, 1.89) (#) | 1.63 (1.43, 1.85) | 1.07 (0.84, 1.35) |
| 16 to 18 months after | 0.90 (0.80, 1.01) | 1.64 (1.57, 1.71) (#) | 1.47 (1.28, 1.68) | 0.94 (0.73, 1.21) |
| 19 to 21 months after | 0.76 (0.67, 0.87) | 1.54 (1.47, 1.61) (#) | 1.47 (1.28, 1.69) | 0.92 (0.71, 1.19) |
| 22 to 24 months after | 0.58 (0.50, 0.66) | 1.46 (1.39, 1.53) (#) | 1.52 (1.32, 1.75) | 0.97 (0.75, 1.25) |
|  |  |  |  |  |
| ***Death of Child*** |  | | | |
| *Accidents (ICD-10: S, T)* | Expected difference between index group and controls, OR (95% CI) | | | |
| Index vs controls | 1.36 (0.95, 1.94) | 1.30 (0.97, 1.75) |  |  |
|  | Additional risk increases in index group compared to controls, OR (95% CI) | | | |
| 22 to 24 months before | 1 | 1 |  |  |
| 19 to 21 months before | 1 | 1 |  |  |
| 16 to 19 months before | 1 | 1 |  |  |
| 13 to 16 months before | 1 | 1 |  |  |
| 10 to 13 months before | 1.14 (0.66, 1.98) | 1.14 (0.72, 1.81) |  |  |
| 7 to 10 months before | 1.37 (0.82, 2.30) | 0.89 (0.53, 1.48) |  |  |
| 4 to 7 months before | 0.88 (0.48, 1.61) | 0.69 (0.39, 1.21) |  |  |
| Up to 3 months before | 1.59 (0.97, 2.62) | 1.13 (0.71, 1.82) |  |  |
| Up to 3 monthe after | 54.12 (38.42, 76.25) | 4.84 (3.45, 6.78) |  |  |
| 4 to 6 months after | 18.64 (12.87, 26.99) | 5.00 (3.53, 7.09) |  |  |
| 7 to 9 months after | 11.77 (7.92, 17.51) | 4.73 (3.28, 6.82) |  |  |
| 10 to 12 months after | 8.90 (5.83, 13.59) | 4.23 (2.88, 6.23) |  |  |
| 13 to 15 months after | 6.53 (4.15, 10.26) | 3.67 (2.44, 5.52) |  |  |
| 16 to 18 months after | 5.37 (3.32, 8.67) | 3.30 (2.14, 5.07) |  |  |
| 19 to 21 months after | 4.08 (2.45, 6.81) | 3.29 (2.10, 5.15) |  |  |
| 22 to 24 months after | 3.00 (1.73, 5.18) | 2.90 (1.81, 4.64) |  |  |
|  |  |  |  |  |
| *Not Accidents* | Expected difference between index group and controls, OR (95% CI) | | | |
| Index vs controls | 2.12 (1.78, 2.52) | 1.66 (1.42, 1.94) |  |  |
|  | Additional risk increases in index group compared to controls, OR (95% CI) | | | |
| 22 to 24 months before | 1 | 1 |  |  |
| 19 to 21 months before | 1 | 1 |  |  |
| 16 to 19 months before | 1 | 1 |  |  |
| 13 to 16 months before | 1 | 1 |  |  |
| 10 to 13 months before | 1.21 (0.96, 1.54) | 0.98 (0.77, 1.25) |  |  |
| 7 to 10 months before | 1.15 (0.90, 1.47) | 0.85 (0.66, 1.09) |  |  |
| 4 to 7 months before | 1.24 (0.98, 1.58) | 0.92 (0.72, 1.18) |  |  |
| Up to 3 months before | 2.55 (2.09, 3.12) | 1.18 (0.94, 1.47) |  |  |
| Up to 3 monthe after | 30.22 (25.63, 35.65) (*) | 4.37 (3.68, 5.18) |  |  |
| 4 to 6 months after | 11.89 (9.9, 14.27) | 4.63 (3.89, 5.52) |  |  |
| 7 to 9 months after | 6.47 (5.29, 7.92) (*) | 3.59 (2.97, 4.33) |  |  |
| 10 to 12 months after | 4.89 (3.93, 6.08) | 2.96 (2.42, 3.62) |  |  |
| 13 to 15 months after | 3.51 (2.76, 4.45) | 2.26 (1.82, 2.80) |  |  |
| 16 to 18 months after | 2.29 (1.76, 2.98) (*) | 2.01 (1.60, 2.52) |  |  |
| 19 to 21 months after | 1.97 (1.49, 2.60) | 1.77 (1.39, 2.25) |  |  |
| 22 to 24 months after | 1.92 (1.43, 2.57) | 1.45 (1.12, 1.87) |  |  |
| Odds ratios are all set to unity for the first 4 observation periods which were used to predict the results for case subjects had they not experienced the death of a close relative. To indicate significant differences between younger and older relatives, accidents/not accidents, we added, in the table for younger/accidents, (*) representing p<0.01 and (#) representing p<0.001 | | | | |

Table 7

Rates of ICD-10 Subforms of Stress Reactions in Individuals Exposed versus Unexposed to Death of a Sibling (F430 – acute stress reaction, F431 - Post-traumatic stress disorder, F432 - Adjustment disorders, F438 - Other reactions to severe stress and F439 - Reaction to severe stress, unspecified)

.

|  | *Women (N = 181,924)* | | | *Men (N = 178,016)* | | |
| --- | --- | --- | --- | --- | --- | --- |
|  | With diagnosis | | | With diagnosis | | |
|  | Exposed | Unexposed | OR (95% CI) | Exposed | Unexposed | OR (95% CI) |
| SR, before | 4,544 (2.50%) | 3,467 (1.91%) | 1.41 (1.25, 1.58) | 1,682 (0.94%) | 1,267 (0.71%) | 1.33 (1.24, 1.43) |
| SR, after | 7,497 (4.12%) | 3,558 (1.96%) | 2.15 (2.07. 2.34) | 3,000 (1.69%) | 1,280 (0.72) | 2.37 (2.21, 2.53) |
|  |  |  |  |  |  |  |
| F430, before | 688 (0.38%) | 489 (0.27%) | 1.41 (1.25, 1.58) | 357 (0.20%) | 250 (0.14%) | 1.43 (1.22, 1.68) |
| F430, after | 1441 (0.79%) | 532 (0.29%) | 2.72 (2.46, 3.01) | 739 (0.42%) | 298 (0.17%) | 2.49 (2.17, 2.84) |
|  |  |  |  |  |  |  |
| F431, before | 181 (0.10%) | 167 (0.09%) | 1.08 (0.87, 1.34) | 87 (0.05%) | 48 (0.03%) | 1.81 (1.27, 2.58) |
| F431, after | 293 (0.16%) | 173 (0.10%) | 1.69 (1.40, 2.05) | 106 (0.06%) | 45 (0.03%) | 2.36 (1.66, 1.34) |
|  |  |  |  |  |  |  |
| F432, before | 226 (0.12%) | 201 (0.11%) | 1.12 (0.93, 1.36) | 136 (0.08%) | 97 (0.05%) | 1.40 (1.08, 1.82) |
| F432, after | 354 (0.19%) | 195 (0.11%) | 1.82 (1.53, 2.16) | 208 (0.12%) | 109 (0.06%) | 1.90 (1.51, 2.40) |
|  |  |  |  |  |  |  |
| F438, before | 551 (0.30%) | 508 (0.28%) | 1.08 (0.96, 1.22) | 176 (0.10%) | 150 (0.08%) | 1.17 (0.94, 1.46) |
| F438, after | 762 (0.42%) | 652 (0.36%) | 1.17 (1.05, 1.30) | 252 (0.14%) | 204 (0.11%) | 1.24 (1.03, 1.49) |
|  |  |  |  |  |  |  |
| F439, before | 3,218 (1.77%) | 2,397 (1.32%) | 1.35 (1.28, 1.42) | 1,037 (0.58%) | 818 (0.46%) | 1.27 (1.16, 1.39) |
| F439, after | 5,428 (2.98%) | 2,325 (1.28%) | 2.38 (2.26, 2.50) | 1,942 (1.09%) | 754 (0.42%) | 2.59 (2.39, 2.82) |

Figures for Incident and Recurrent Cases of SR, MD, AUD and DUD – *Note Differences in the Rates Depicted on the Y-Axis*

Figure 1a Parents


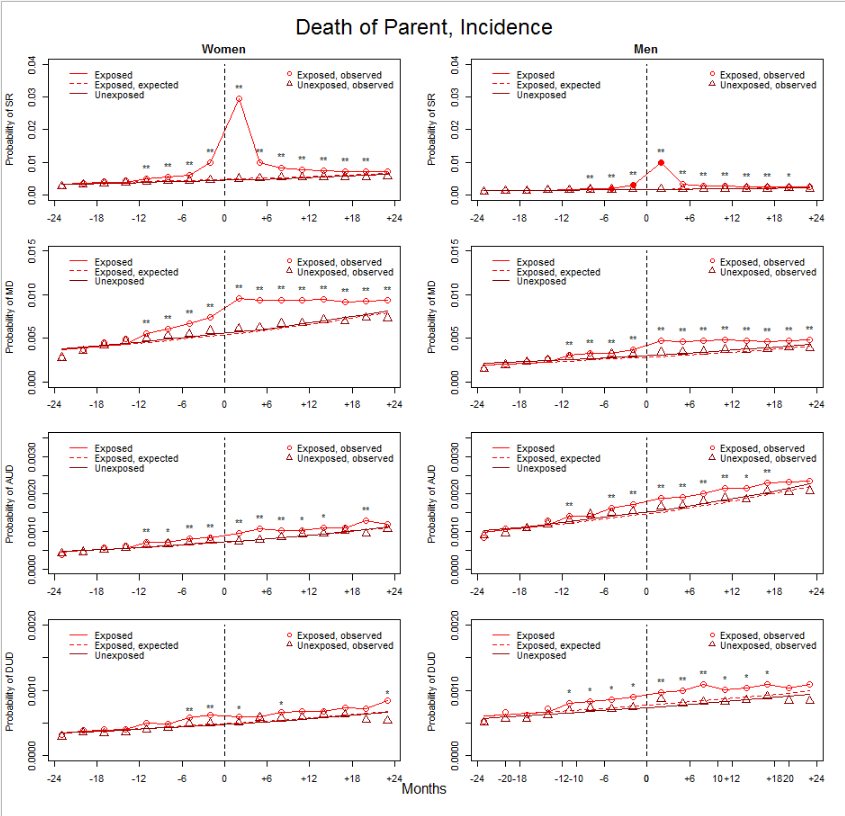


Figure 1b Parents


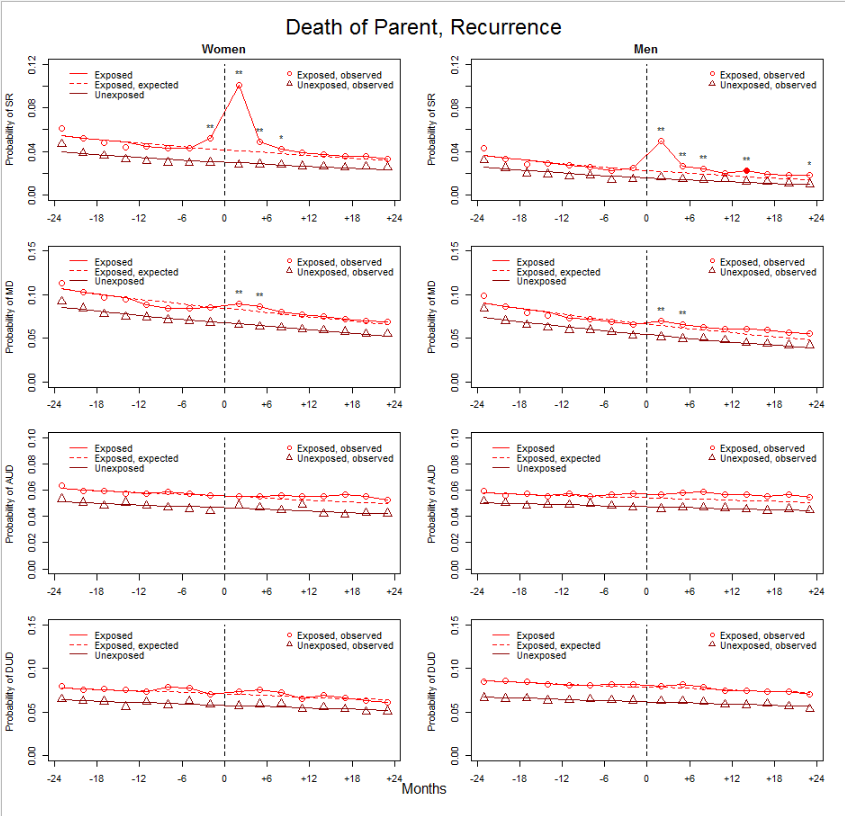


Figure 2a Siblings


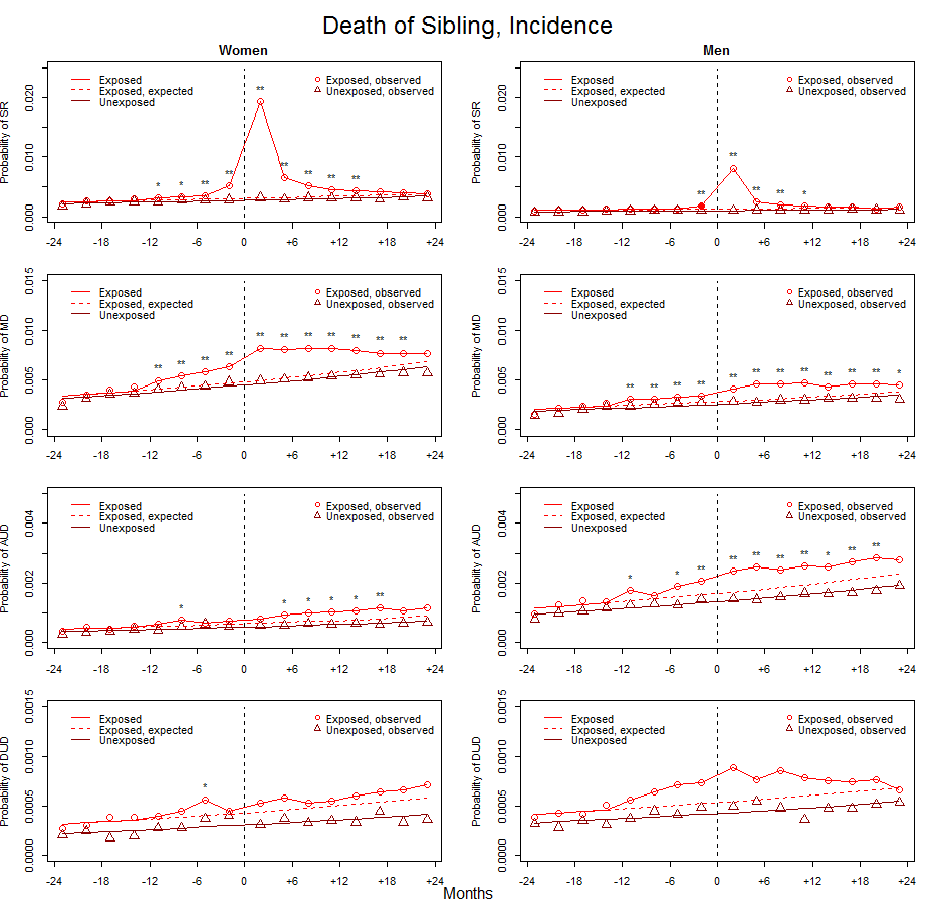


Figure 2a Siblings


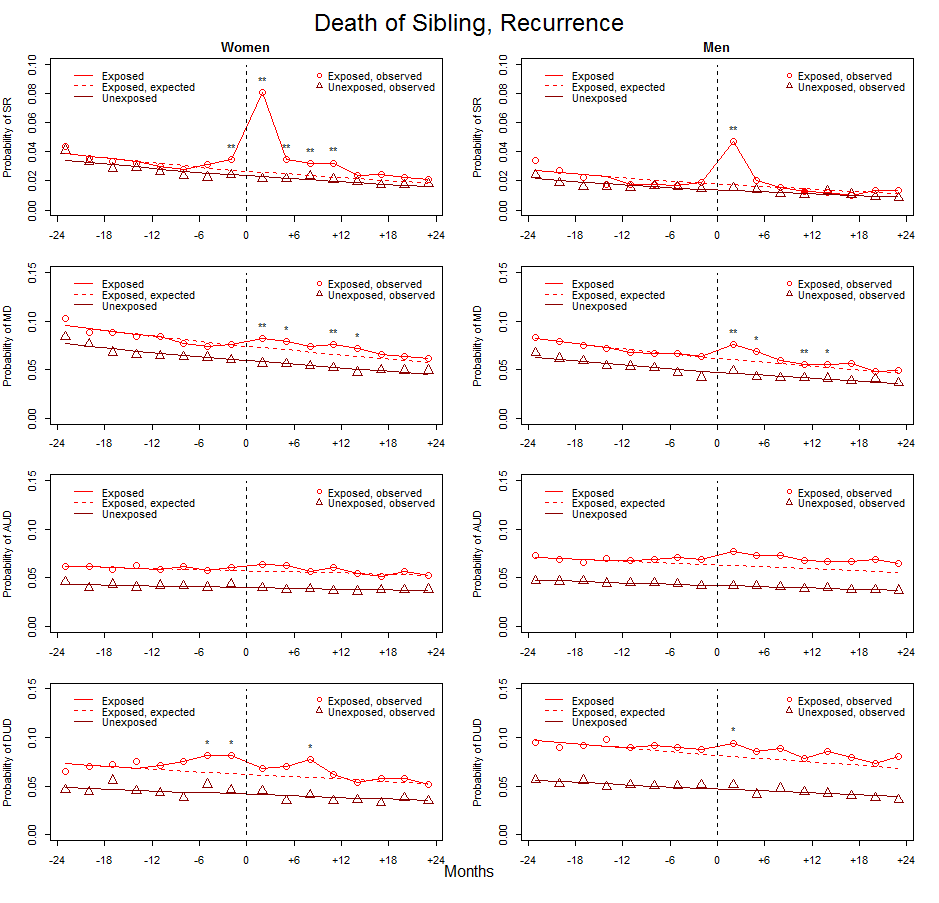


Figure 3a Spouses


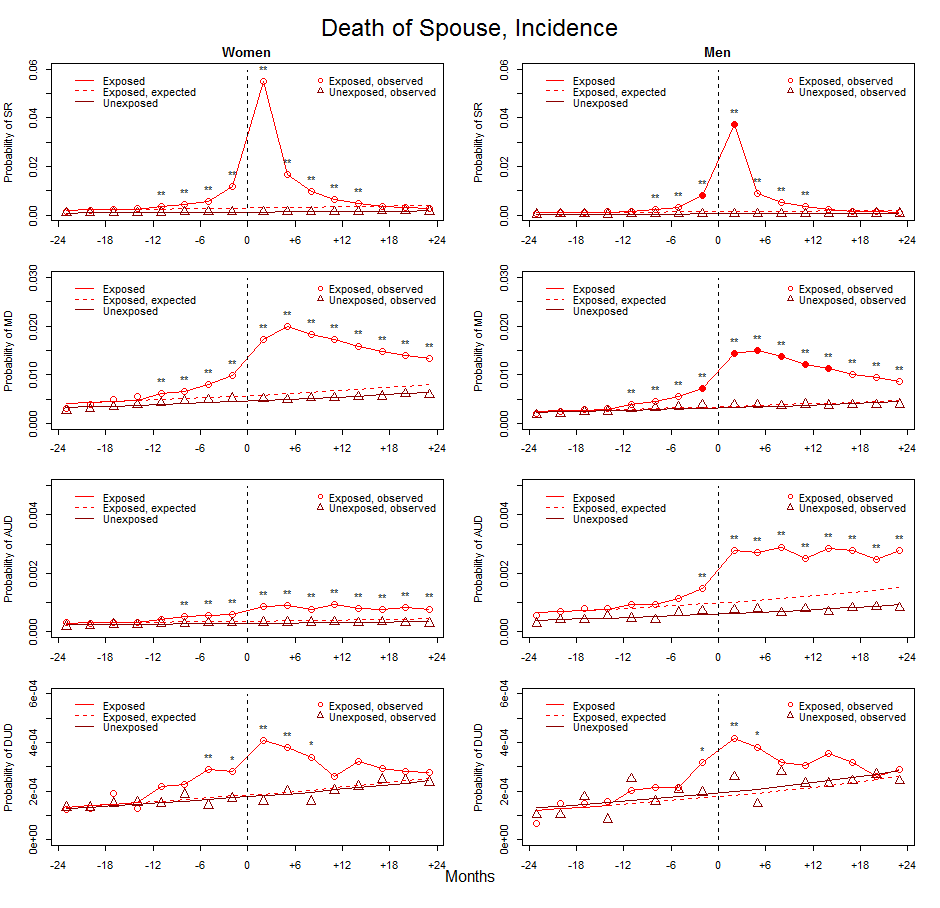


Figure 3b Spouses


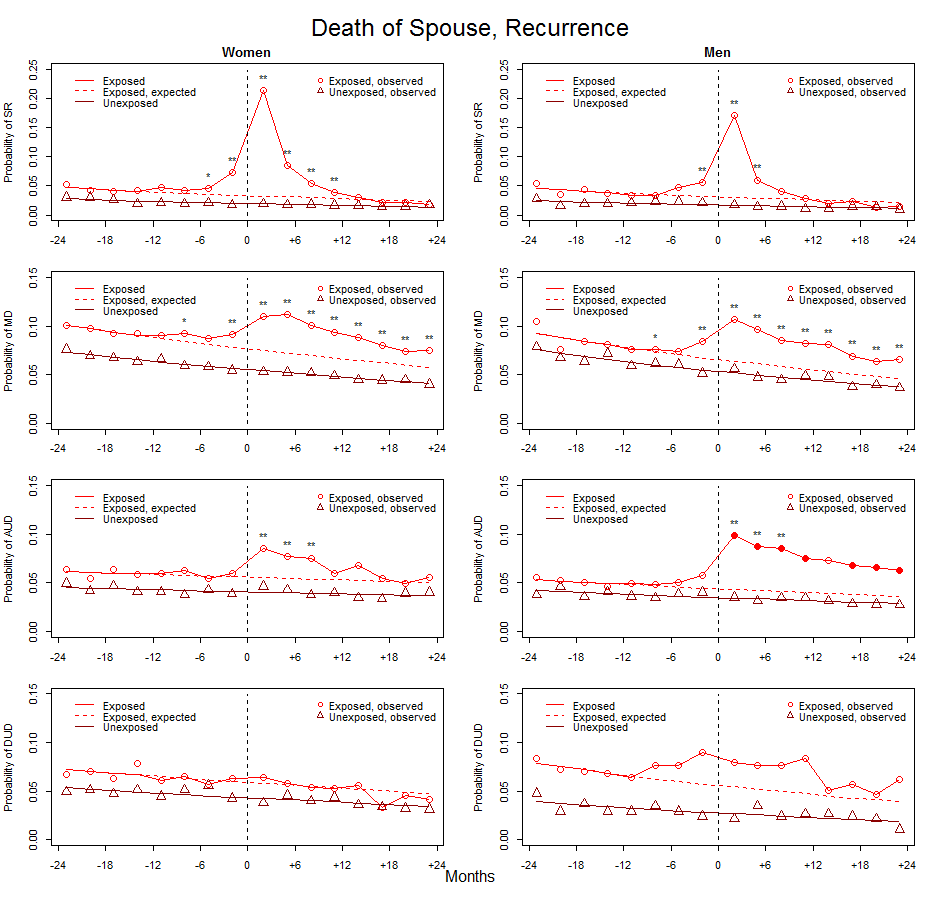


4a – Child


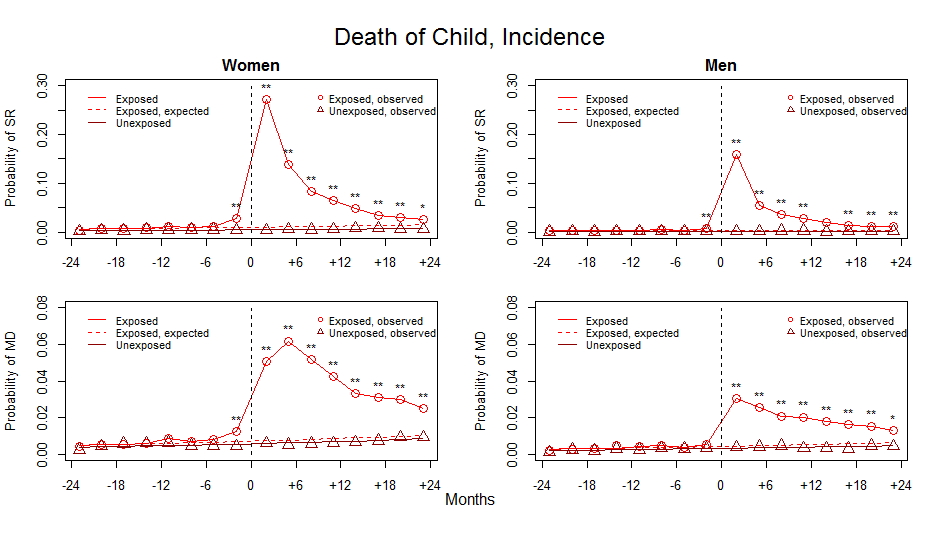


4b – Child


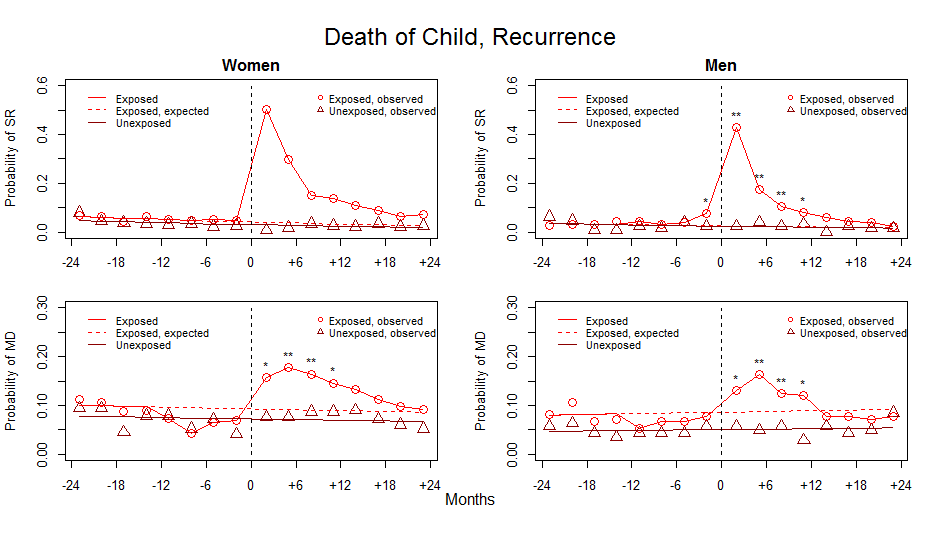


1. Sjölander A, Greenland S. Ignoring the matching variables in cohort studies–when is it valid and why? Statistics in medicine. 2013;32:4696-4708.
